# Biofuel production: exploring renewable energy solutions for a greener future

**DOI:** 10.1186/s13068-024-02571-9

**Published:** 2024-10-15

**Authors:** R. El-Araby

**Affiliations:** Chemical Engineering and Pilot Plant Department, Institute of Engineering Research and New and Renewable Energy, National Research Centre, Cairo, Egypt

**Keywords:** Biofuels, Renewable fuels, Biomass, Algae biofuels, Biofuel feedstocks, Sustainable energy, Biofuel technologies

## Abstract

Biofuel production has emerged as a leading contender in the quest for renewable energy solutions, offering a promising path toward a greener future. This comprehensive state-of-the-art review delves into the current landscape of biofuel production, exploring its potential as a viable alternative to conventional fossil fuels. This study extensively examines various feedstock options, encompassing diverse sources such as plants, algae, and agricultural waste, and investigates the technological advancements driving biofuel production processes. This review highlights the environmental benefits of biofuels, emphasizing their capacity to significantly reduce greenhouse gas emissions compared to those of fossil fuels. Additionally, this study elucidates the role of biofuels in enhancing energy security by decreasing reliance on finite fossil fuel reserves, thereby mitigating vulnerabilities to geopolitical tensions and price fluctuations. The economic prospects associated with biofuel production are also elucidated, encompassing job creation, rural development, and the potential for additional revenue streams for farmers and landowners engaged in biofuel feedstock cultivation. While highlighting the promise of biofuels, the review also addresses the challenges and considerations surrounding their production. Potential issues such as land use competition, resource availability, and sustainability implications are critically evaluated. Responsible implementation, including proper land-use planning, resource management, and adherence to sustainability criteria, is emphasized as critical for the long-term viability of biofuel production. Moreover, the review underscores the importance of ongoing research and development efforts aimed at enhancing biofuel production efficiency, feedstock productivity, and conversion processes. Technological advancements hold the key to increasing biofuel yields, reducing production costs, and improving overall sustainability. This review uniquely synthesizes the latest advancements across the entire spectrum of biofuel production, from feedstock selection to end-use applications. It addresses critical research gaps by providing a comprehensive analysis of emerging technologies, sustainability metrics, and economic viability of various biofuel pathways. Unlike previous reviews, this work offers an integrated perspective on the interplay between technological innovation, environmental impact, and socio-economic factors in biofuel development, thereby providing a holistic framework for future research and policy directions in renewable energy.

## Introduction

### Growing concerns about climate change and fossil fuel depletion

**Fig. 1:**
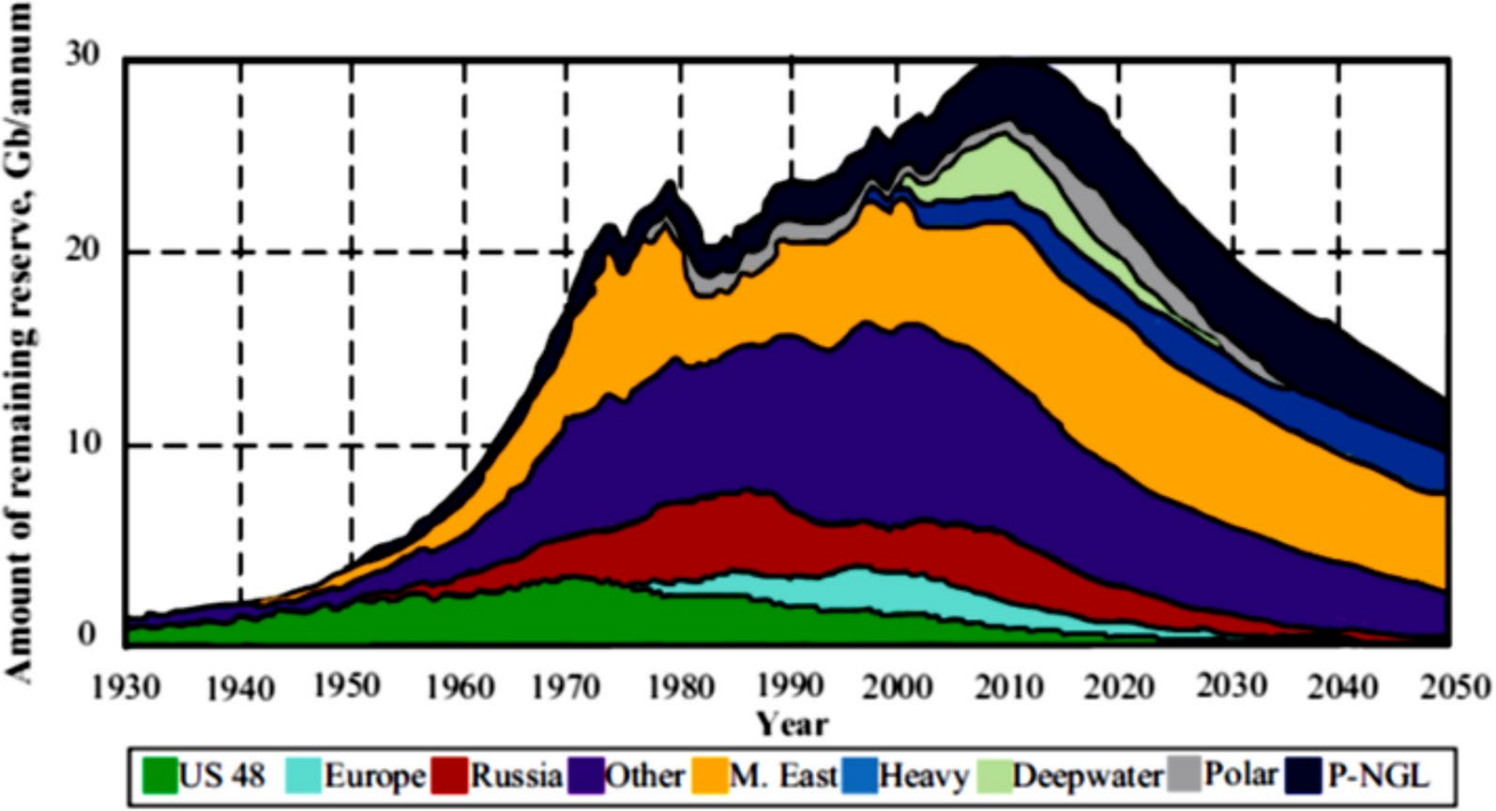
Hubbert’s curve for the amount of fossil fuel reserves [72]

Conventional fossil fuel energy resources such as coal, natural gas and oil are limited in quantity and will eventually be depleted at current global production rates, with estimated reserve-to-production lifetimes of approximately 139, 48.8 and 53.5 additional years, respectively [77, 165]. As depicted in Fig. 1, fossil fuel production over time follows Hubbert's curve, gradually increasing to a peak and then diminishing as reserves decline, marking their end. Hubbert's curve, also known as the Hubbert peak theory, is a influential concept in resource depletion modeling, originally developed by geologist M. King Hubbert in 1956. The theory posits that the production rate of a finite resource, such as fossil fuels, follows a bell-shaped curve over time. According to this model, production initially increases exponentially, reaches a peak when approximately half of the resource has been extracted, and then gradually declines as the resource becomes more difficult and expensive to extract. While originally applied to oil production, Hubbert's curve has since been adapted to model the production and depletion of various finite resources, including some renewable resources that are exploited faster than they can regenerate.

However, estimates could change based on production, consumption and discoveries. Nonetheless, affordable fossil fuels will eventually run out, compelling a shift to renewables [62, 63]. This is why advancing renewable technologies is now important. Unlike fossil fuels, which take millions of years to form, at present usage rates, the fossil fuel era will last only approximately 400 years [84]. Investing in renewables is thus crucial, as future generations will witness the end of finite fossil fuels.

Fossil fuel combustion releases greenhouse gases such as CO_2_ and methane into the atmosphere, causing climate change [132]. Fossil fuel reserves are finite and being rapidly depleted, with oil potentially peaking soon after [154]. These interlocking climate and energy crises require an urgent transition to renewable energy for sustainability and emission reduction [14, 154]. Projections show that renewable electricity demand will increase substantially by 2030. According to the International Energy Agency (IEA), global renewable electricity capacity is expected to rise by over 60% from 2020 to 2026, reaching more than 4800 GW. This is equivalent to the current total global power capacity of fossil fuels and nuclear combined. Specifically, renewable electricity generation is projected to grow by 8% annually on average, reaching 12,350 TWh by 2026 [67]. Furthermore, the International Renewable Energy Agency (IRENA) forecasts that the share of renewable energy in global electricity generation could increase from 26% in 2018 to 57% by 2030 in their Transforming Energy Scenario [68]. However, energy poverty still hinders access to clean energy globally. The environmental crisis has prompted the striving for carbon neutrality per the UN Sustainable Development Goals [14], including Goal 7 of transitioning from fossil fuels by 2030 [92–94]. Human activities such as fossil fuel burning and CFC chlorofluorocarbons refrigerant use have scientifically undisputed impacts CO_2_, methane and CFCs, respectively cause global warming, ozone depletion and both, respectively [153]. With increasing awareness, fossil fuel use will hopefully be reduced, and reserves preserved for transitional purposes, as renewable energy will ultimately remain when fossil fuels run out [62, 63].

### Importance of renewable energy solutions

Renewable energy is obtained from naturally replenished sources, including sunshine, wind, water, geothermal heat, and biomass, which are available in endless numbers, as opposed to from finite fossil and nuclear fuels [6]. While many renewable energy sources, such as solar and wind power, produce minimal direct emissions during operation, the environmental impact of renewables can vary depending on the specific technology and lifecycle considerations [6, 142]. For instance, some biomass conversion processes do produce greenhouse gas emissions, albeit generally at lower levels compared to fossil fuel processes [76]. However, the overall environmental benefits of renewables are significant when compared to conventional fossil fuel energy sources. Renewables generally have a much lower carbon footprint over their lifecycle, contribute to improved air quality, and offer sustainable long-term energy solutions [69].

. They provide a reliable long-term energy supply, reduce dependence on volatile fossil fuel imports, spur job growth and innovation, yield cost savings, and enable decentralized resilient power [6]. Prominent renewable technologies include solar photovoltaics, wind turbines, hydropower plants, biomass plants combusting organic material, geothermal plants, tidal/wave systems, and hydrogen fuel cells electrochemically converting hydrogen to electricity [142]. Renewables thus ensure clean, sustainable energy security.

### Promising alternative biofuel

Biofuels from renewable biomass, such as plants and organic waste, provide a sustainable alternative to mitigate fossil fuel depletion and climate change [32], Pandey et al.,2018). Biofuels can be classified into different generations based on their feedstock and production technology. Conventional or first-generation biofuels are derived from food crops such as corn, sugarcane, and vegetable oils. Second-generation biofuels use nonfood biomass like agricultural residues, wood, and municipal waste. Third-generation biofuels, which include dedicated energy crops and algae, represent more advanced and sustainable options [32]. Advanced conversion methods continuing to overcome economic and technological obstacles to enable wider utilization. Biofuels can blend with conventional fuels or directly substitute them with existing infrastructure, allowing a smooth adoption transition without substantial system changes [38]. Questions remain about the optimal feedstocks and processes for maximizing greenhouse gas reductions versus conventional fuels. Exploring progress and challenges across biofuel types and technologies provides insights into the sustainable solutions that different biofuels can provide through continued advancement, contributing to a greener future [126].

This comprehensive review addresses several key research gaps in the field of biofuel production. Firstly, it provides an up-to-date synthesis of advancements across various biofuel generations, offering insights into the most promising pathways for future development. Secondly, it critically evaluates the sustainability and economic viability of different biofuel technologies, an area often overlooked in technical reviews. Thirdly, it explores the potential of emerging feedstocks and conversion technologies that have not been extensively covered in previous literature. By integrating technological, environmental, and socio-economic perspectives, this review aims to provide a holistic understanding of the challenges and opportunities in biofuel production, serving as a valuable resource for researchers, policymakers, and industry stakeholders working towards a sustainable energy future.

## The imperative for renewable energy

Fossil fuels meet ~ 80% of energy needs but increasingly demonstrate negative environmental impacts from air pollution due to climate change, alongside dwindling finite supplies and volatile prices [49, 62, 63]. The pressing need for clean, renewable energy alternatives to power society has thus never been met. Renewables such as solar, wind, geothermal and biofuel can provide substantial emission-free energy for electricity and transportation, supporting energy independence and stability through zero fuel costs. An accelerated transition is attainable through supportive policies, infrastructure investment, and efficiency improvements to increase adoption. With bold action and innovation, renewables can transform economies, environments and quality of life for current and future generations [62, 63].

### Environmental impact of fossil fuel consumption

The use of fossil fuels such as coal, oil, and gas have major environmental and health externalities not reflected in market prices, including air/water pollution, crop/forest/wildlife damage, and greenhouse gas emissions driving climate change and ocean acidification. Ocean acidification refers to the ongoing decrease in the pH of the Earth’s oceans, primarily caused by the absorption of atmospheric carbon dioxide (CO_2_). As CO_2_ levels in the atmosphere increase due to human activities, more CO_2_ dissolves in seawater, forming carbonic acid. This process leads to a reduction in seawater pH, decreased carbonate ion concentration, and lower calcium carbonate mineral saturation states. Extreme weather events costing billions of dollars are linked to climate change from fossil fuel burning. Sea level rise, increasing flooding, storm surges, and saltwater intrusion are other concerns (Fact-sheet 2021). Saltwater intrusion is the movement of saline water into freshwater aquifers or surface water systems, typically occurring in coastal areas. This phenomenon is often caused by over-extraction of groundwater, sea-level rise, and changes in weather patterns affecting freshwater recharge.

As freshwater is removed from an aquifer faster than it is replenished, saltwater from the ocean moves inland to fill the void. This process can contaminate freshwater supplies, making them unsuitable for drinking or irrigation, reduce crop yields and damage soil quality in coastal farming areas, and pose long-term challenges for water resource management, as reversing saltwater intrusion can be difficult and costly. Climate change, particularly rising sea levels, is expected to exacerbate saltwater intrusion in many coastal regions worldwide.

**Fig. 2:**
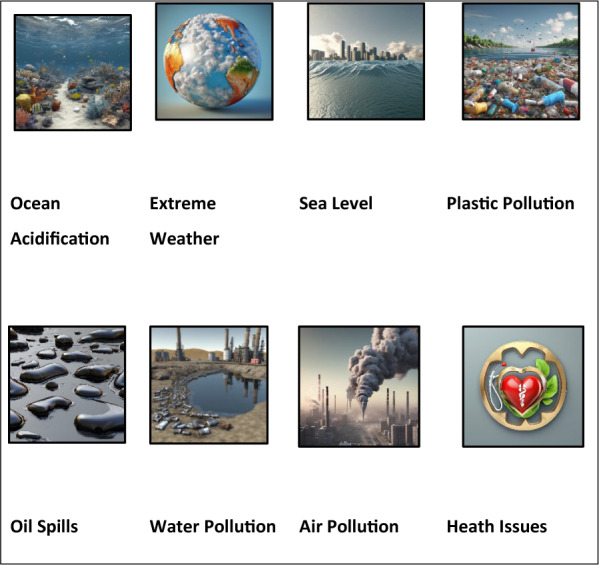
Impacts of fossil fuel usage

Additional issues, such as oil spills and acid mine drainage from coal, further highlight the environmental toll of fossil fuels. Reducing dependence on these products is thus crucial for mitigating climate change and protecting the environment overall [73]. Figure 2 shows the different environmental impacts of fossil fuel.

The fossil fuel industry leases vast lands for infrastructure, devastating landscapes, habitats and ecosystems—strip mining razes forests and mountaintops that can never regenerate. Fragmentation destroys critical wildlife breeding and migration habitats, displacing animals into inferior areas to compete with existing wildlife [73]. The extraction and transport of coal, oil and gas also threatens water systems through toxic runoff, spills, fracking fluids or wastewater overflows contaminating rivers, lakes, aquifers and oceans, with pollutants linked to cancer, birth defects and neurological damage [56, 73]. Vast volumes of drilling and mining toxic wastewater are stored in leakage pits and wells. Additionally, fossil fuel-powered transport releases smog-forming pollutants, causing respiratory illness from sustained exposure [56].

### Increasing energy demand

**Fig. 3:**
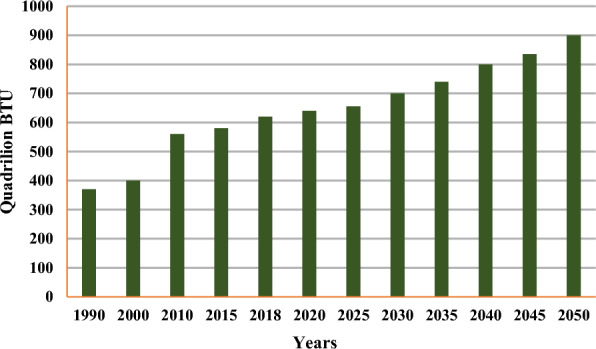
Worldwide energy consumption from 1990 to 2050 [26]

Projecting future renewable energy demand involves uncertainties around population, economic growth, sectoral/behavioral shifts, technological development, and climate change impacts, all of which can alter energy needs over time [128, 164]. Characterizing these complex interaction demand uncertainties for different energy sources can inform policymakers' long-term plans. Leveraging the latest socio-economic and climate scenarios allows us to empirically assess demands by fuel type across projections [164]. This integrated uncertainty analysis approach aims to accelerate renewable adoption despite future demand uncertainties. Overall, global energy demand is projected to increase nearly 50% by 2050 compared to 2020, with developing nations driving almost half of this growth (Fig. 3) as rising populations and GDP improve living standards and energy access [26, 164].

**Fig. 4:**
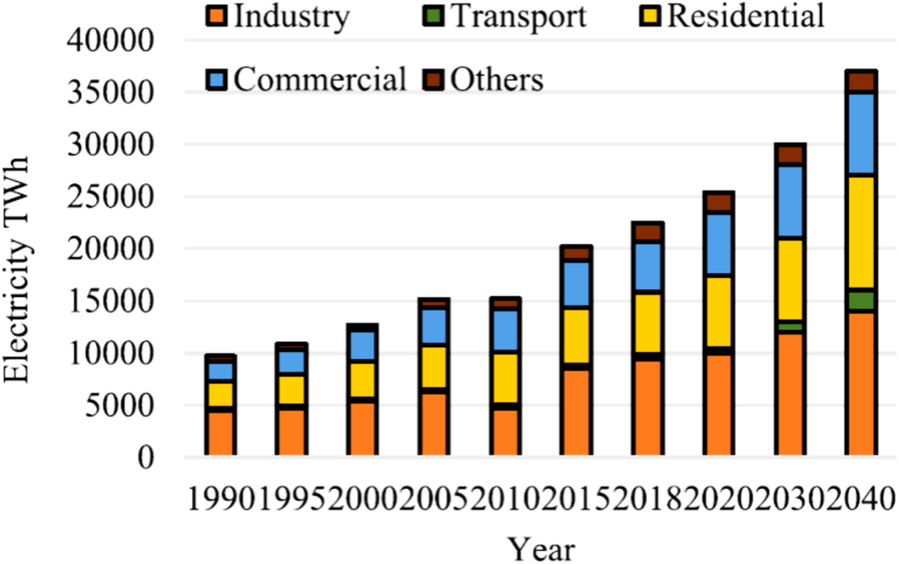
Electricity consumption by sector over time (from 1990 to 2040) [26]

Historical data show that the industrial sector leads in electricity usage, followed by residential use. Recently, transportation electricity demand has been rising due to increasing electrification, with forecasts showing this trend intensifying. Overall electricity usage is predicted to rise by almost 1.5 times by 2040 versus the current level (Fig. 4). Specifically, the U.S. Energy Information Administration (EIA) predicts over double the electricity demand in 2050 relative to that in 2018 [26]. While industry currently consumes the most electricity, transportation electrification is driving rapid demand increases, with total electricity usage expected to increase 50–100% in the coming decades across all sectors.

**Fig. 5:**
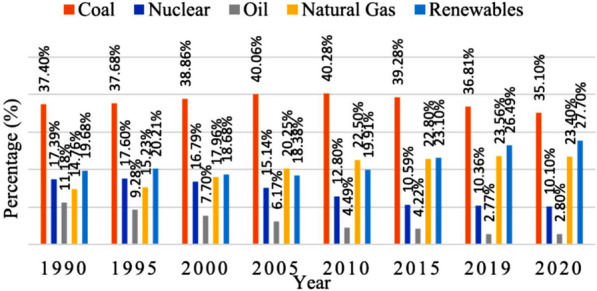
Comparison of fossil fuel and renewable generation increases [26]

**Fig. 6:**
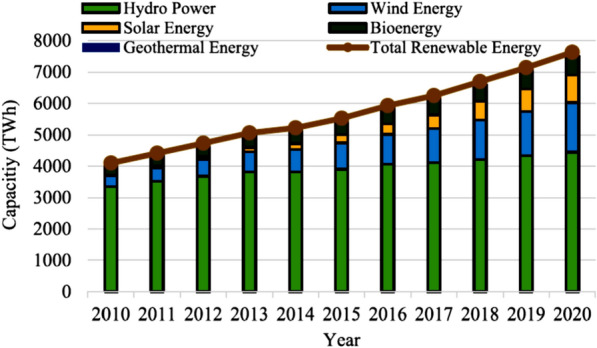
Total renewable energy production (2010–2020) [26]

Currently, most of the electricity is from fossil fuels, but renewables such as solar, wind, hydro and biomass are being generated. The data show that the share of total generation of renewable energy has grown significantly since 2010, while that of fossil fuels has declined (Fig. 5). Projections estimate that renewables will expand from a 23% share in 2015 to 63% by 2050. From 2010 to 2020, the gap between fossil fuels and renewables narrowed, with fossil fuel generation growing slightly and then dropping in 2020 due to COVID-19, while renewables rose steadily throughout, surpassing fossils in 2020. Overall, between 2010 and 2020, the consumption of renewables for electricity generation substantially increased from 4098 to 7627 TWh (Fig. 6), illustrating the growing utilization of renewable energy over the decade [26]. Trends point to renewables steadily displacing fossil fuel electricity generation to potentially account for more than 60% by mid-century.

**Fig. 7:**
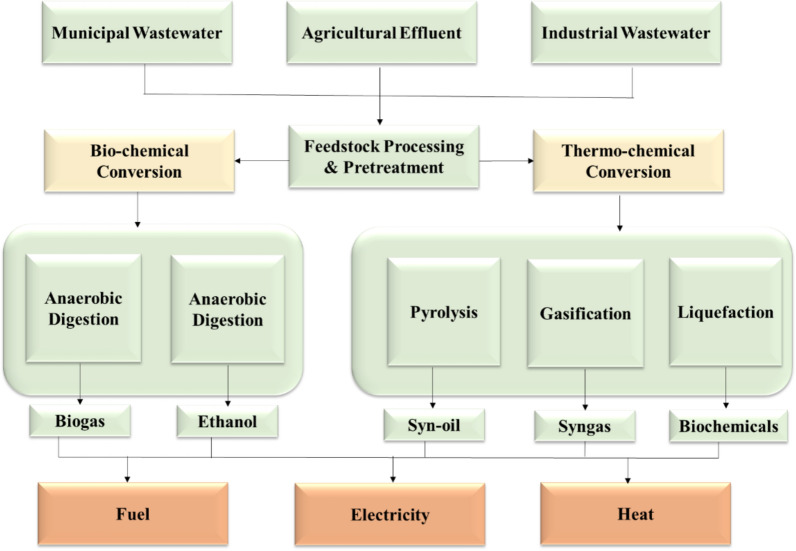
Converting industrial and household waste into three key products: biofuels, thermal energy, and electrical power

Another type of alternative energy is biomass energy, which may be used in chemical processes such as combustion, pyrolysis, or gasification to create heat (biochar), tar (biofuel), and syngas (biogas). The product can then be utilized to generate power using specialized techniques. The procedures for generating power from biomass are shown in Fig. 7 [26].

### Role of renewable energy in reducing carbon emissions

Fossil fuels were the primary energy source for several years before renewable options emerged. Coal and oil were more widely used than natural gas initially. With growing environmental concerns, research has focused on shifting utilization from fossils to renewables [147]. While fossil fuel-dominated energy production has historically prevailed, renewable usage, especially geothermal, biomass and hydro fuel, began rising approximately 1965, with wind and solar energy growing substantially since the 1990s as awareness of fossil fuel environmental harm increased [156]. Renewables such as solar, wind, hydroelectric and geothermal materials are crucial for combating climate change and providing a cleaner alternative to fossil fuels. Analysis shows that renewables and efficiency can account for more than 90% of needed energy-related emissions reductions [70]. Governments and companies have critical roles in enabling a renewable transition to create a sustainable, low-carbon future.

## Bioenergy: promising solution

Bioenergy is a renewable energy source derived from biomass, which includes organic materials like crops, trees, and algae. These materials can be obtained through various pathways, including photosynthesis, non-photosynthetic processes like animal waste and food waste, secondary utilization of products like used cooking oils, and microbial processes such as biogas generation [25].

Biopower is the energy generated by biomass burning, either alone or in conjunction with coal, natural gas, or other fuels. Direct-fired systems generate high-pressure steam, which powers turbines attached to electric generators. The technical hurdles include feedstock quality, boiler chemistry, ash deposition, and disposal. Cofiring biomass with coal in power stations can assist in renewable energy regulation and minimize pollution [25, 29].

**Fig. 8:**
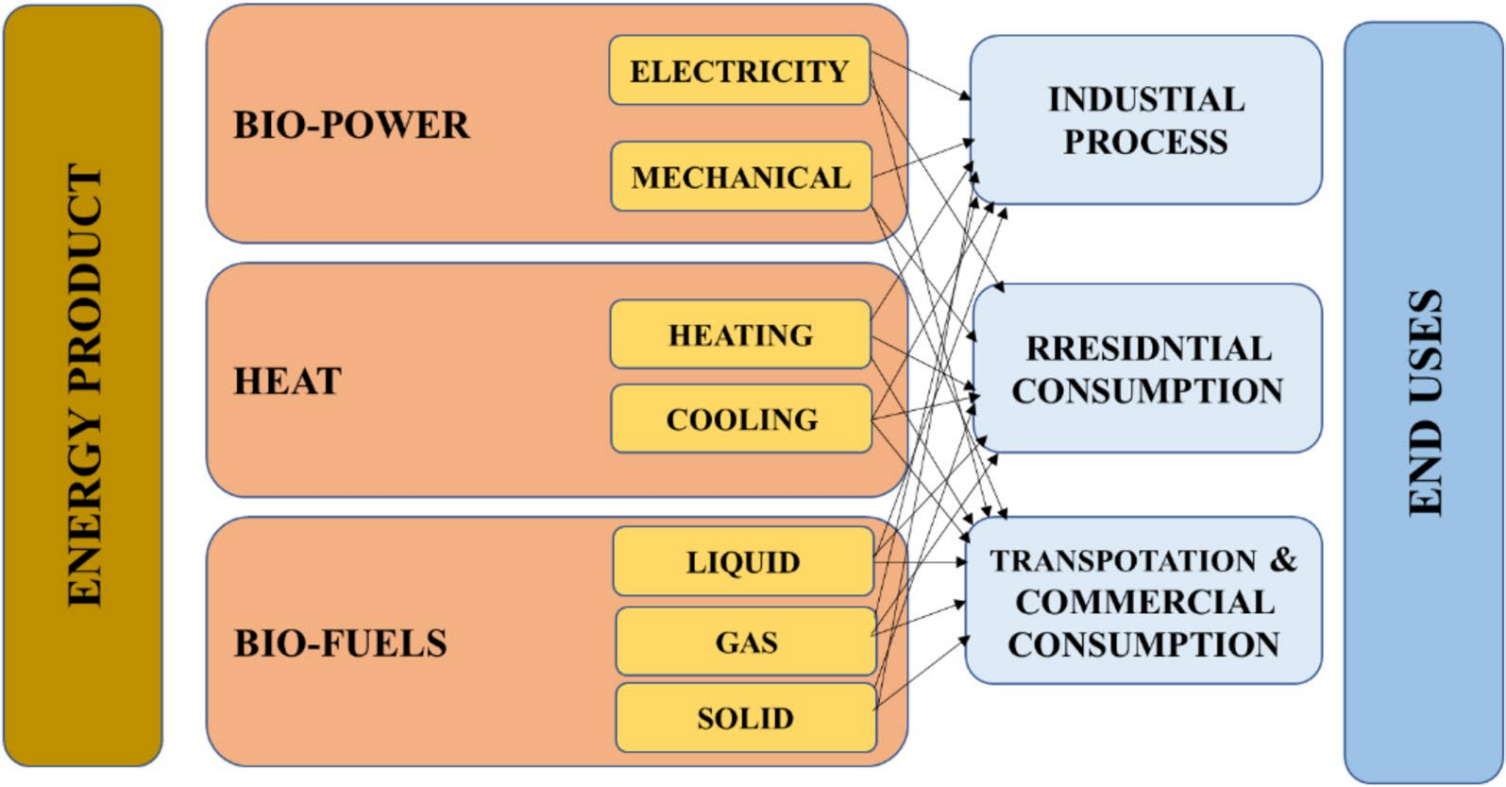
Applications of bioenergy

Biofuel includes solid, liquid, and petrol fuels. Solid fuels are commonly employed for space heating through burning. Liquid and petrol fuels are utilized in transportation and industrial activities. Bioethanol and biodiesel are two important types of biofuels. Biofuel is regarded as an effective alternative source of energy due to its valuable qualities, which include minimal greenhouse gas emissions, biodegradability, and nontoxicity [25, 115]. Figure 8 illustrates the energy products and their end uses.

Burning bioenergy produces a considerable amount of CO_2_ and other greenhouse gases. However, this process produces the same amount of CO_2_ as the plant acquired during photosynthesis when living. As a result, bioenergy is a carbon neutral or net zero energy source, which means that it produces and absorbs the same amount of CO_2_. Furthermore, bioenergy is readily available across the world. Therefore, this is an excellent alternative to fossil fuels [62, 63].

### Global biofuel production: focus on GBA and biodiesel in leading countries

**Fig. 9:**
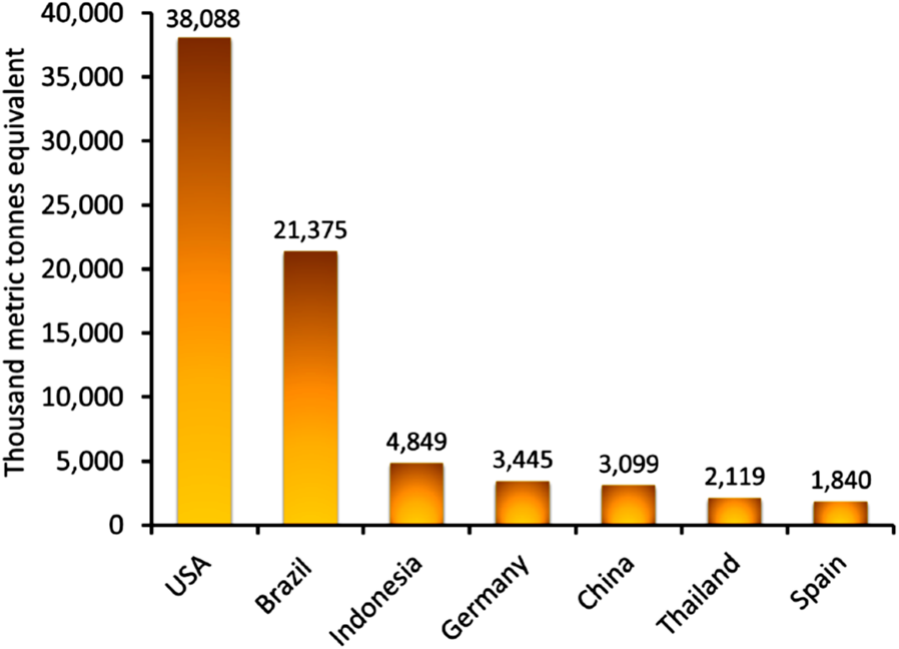
Biodiesel production in leading countries [152]

On September 10, 2023, India-led Global Biofuel Alliance (GBA) members decided to promote biofuel production and use of fossil fuels in transportation and industry to collectively reduce emissions. The multi-stakeholder GBA of governments, organizations and industry aims to accelerate global biofuel uptake. Biofuels from renewable biomass and agricultural waste are considered more sustainable alternatives to fossil fuels with lower carbon emissions. The goal of the GBA is to mainstream these climate-friendly alternatives, greatly contributing to emission reductions [157]. The GBA now has 22-member countries and 12 organizations. As shown in Fig. 9, which shows the top 7 biodiesel producing countries, GBA membership spans major biofuel producers seeking to further grow production and use of these lower-carbon fuels (Microalgae: Global Strategic Business Report 2023).

## Classification of biofuels

**Fig. 10:**
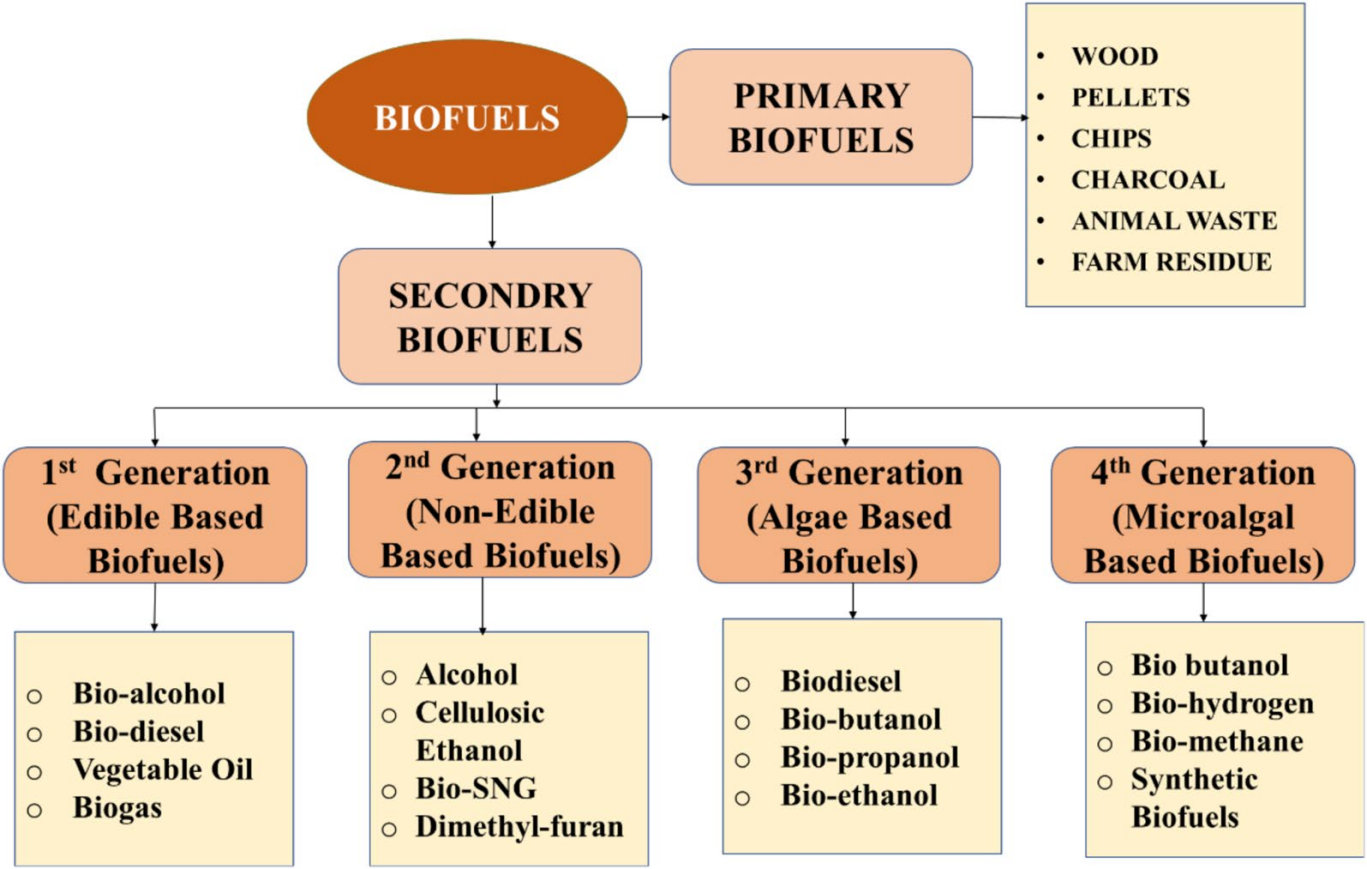
Biofuel types and their generations

Biofuels can be produced in liquid, gaseous or solid forms and are primarily produced from edible crops, cultivated nonfood feedstocks, and agricultural waste. Primary biofuels such as firewood, plants and animal waste are directly used [148]. Secondary biofuels are biomass converted into biodiesel, bioethanol, biohydrogen or biogas. Biobutanol has a longer outlook than bioethanol and can be made more efficient from corn stoves, but substantial feedstocks such as sugarcane bagasse and rice straw may still be used [19, 32]. Biofuels are also categorized by generation based on their feedstock sources, as depicted in Fig. 10: first, generation from food crops, second, from nonfood plants, third, from algae; and fourth, from genetically engineered crops and grasses.

### Classification of biofuels according to feedstock

#### First-generation biofuels

First-generation biofuels are produced from edible feedstocks, such as bioethanol from corn and sugarcane and biodiesel from oil seed crops (soybean, oil palm, rapeseed, and sunflower), using well-understood, economically viable technologies and processes such as fermentation, distillation, and transesterification [32, 106, 136, 148]. First-generation biofuels have a marginal advantage over fossil fuels in terms of greenhouse gas emissions since they take a large amount of energy to create, harvest, and process. However, they present significant challenges including competition with food production, land use concerns, limited greenhouse gas reduction when considering their lifecycle, high water usage, and potential soil degradation from intensive monoculture farming. These disadvantages have prompted increased research into second- and third-generation biofuels to address these concerns while maintaining the benefits of renewable fuel production [143].

#### Second-generation biofuels

The debate over first-generation raw materials for food security has led to a shift toward second-generation lignocellulosic raw materials, derived from nonfood biomass of plants or animals. These materials, such as vegetable grasses and forest residues, are abundant in natural ecosystems and can be used as feedstocks for biofuel production [134]. Lignocellulose-based biofuels have the potential to reduce greenhouse gas emissions, benefit the economy, and enhance energy security. The US and the EU are implementing biotechnology approaches to produce 1.3 billion tons annually without compromising food security [106].

#### Third-generation biofuels

Bioenergy from algae represents the third generation of biofuels. Algae and microorganisms are the primary feedstocks, produced through biochemical and thermochemical conversion processes [16]. They originate from microorganisms that can be cultivated through various methods: autotrophically, using carbon dioxide, light, and nutrients to synthesize biomass, heterotrophically, using organic carbon sources in the absence of light; or mixotrophically, combining both autotrophic and heterotrophic nutrition [118]. These algae are subsequently processed into biofuels [83].

Algal biofuels may be superior replacements for earlier generations due to their faster growth without the need for extensive land or resources [106]. Algae have quicker photosynthesis than land plants used in first/second-generation biofuels. The relevance of algae resides in their noncompetitive nature for food chains while providing diverse end products such as bioethanol, biogas and biodiesel [162]. Various transesterification techniques have been introduced for different microalgae [88]. Transesterification is a crucial process in biodiesel production, involving the reaction of triglycerides with alcohol to produce biodiesel and glycerol. Various techniques such as homogeneous catalysis (base and acid-catalyzed), heterogeneous catalysis, enzymatic transesterification, supercritical alcohol transesterification, ultrasound-assisted transesterification, and microwave-assisted transesterification are used in this process, each with its own advantages and challenges. The choice of technique depends on factors like feedstock quality, production scale, and available resources, with ongoing research focused on optimizing these processes and developing more efficient methods [162]. Due to the utility of biofuel, photosynthetic microorganisms such as algae have recently gained increased academic interest recently [35]. As shown in Table 1, with many algal species, microalgae grow well in diverse water sources with increased lipid and biodiesel energy contents.

**Table 1:** Oil contents of various microalgae contents utilized for biodiesel manufacturing

Species	Oil content (% wt.)
*Chlamydomonas* sp.	22.7
*Chaetoceros muelleri*	13–24
*Parietochloris incise*	062
*Tetraselmis tetrathele*	25–30
*Nostoc commune*	22
*Emiliania huxleyi*	43. 8
*Chroomonas salina*	12–14.5
*Mesotaenium* sp.	19–35
*Spirulina platensis*	4–11
*Synechocystis* sp.	11
*Nannochloris* sp.	25–56
*Neochloris oleoabundans*	35–65
*Chlorella* sp.	28–53
*Schizochytrium* sp.	50–77

#### Fourth-generation biofuels

Fourth-generation biofuels are uncommon and have been under development for several years. It is seen as an extension of the third generation and involves the use of modern biological technology.

**Fig. 11:**
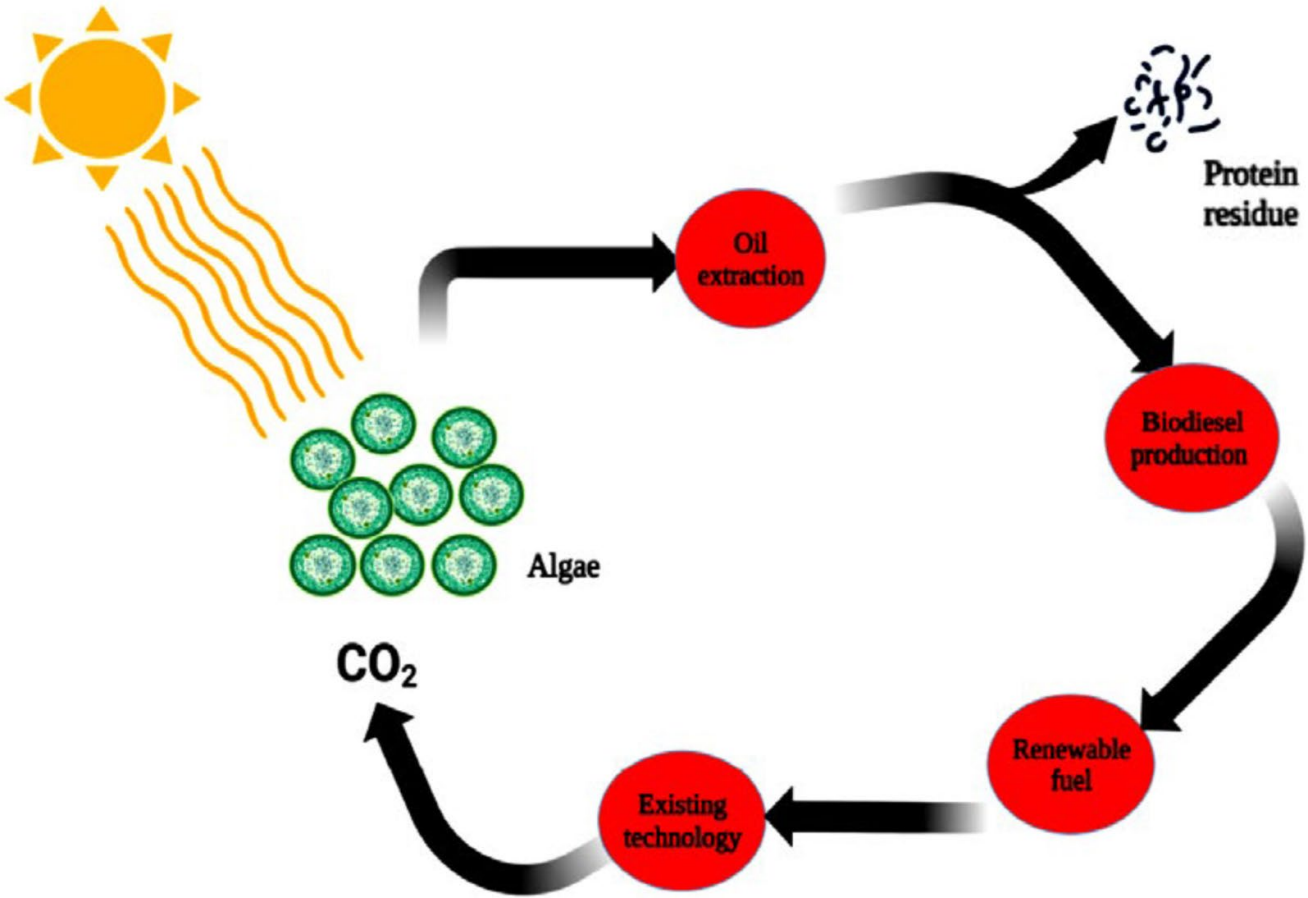
A description of the photosynthesis process in algal chloroplasts for biofuel production [105]

Genetically modified photosynthetic microorganisms (such as cyanobacteria, algae, and fungi) are employed as feedstocks. Photosynthetic bacteria can turn ambient CO2 into ethanol [36]. According to certain related research, some crops capture carbon from the environment and store it in their leaves and stems, after which the carbon is turned into fuel through second-generation procedures [74]. Genetically engineered microbes are utilized in fourth-generation biofuels to increase hydrocarbon output while lowering carbon emissions [33]. Common strategies for the genetic manipulation of algae include enhancing light penetration, increasing photosynthetic efficiency, and decreasing photoinhibition [86]. Notably, metabolic engineering considerably improved the carbohydrate or lipid content in algae (Fig. 11). A comparison of the positive and negative aspects of different generations of biofuel is summarized in Table 2.

**Table 2:** **Comparison of the pros and cons of different generations of biofuels **[1]

Topic	First-generation biofuels	Second-generation biofuels	Third-generation biofuels	Fourth-generation biofuels
Feedstock	Easily accessible vegetables, edible oil**s**, and starch	Nonfood**s** crops	Nonfood crops	Nonfood crops
Land footprint	Arable land	Arable land/forest	Nonarable land	Nonarable land
Major benefits	Simple conversion process	Does not disrupt the food chain supply with lower manufacturing costs	Raw materials can be sourced from inexpensive sources such as waste food oil, garbage, and seawater	Higher lipid content, carbon dioxide absorption, high energy, and faster growth rate
Manufacturing cost	Economically feasible	Economically less productive due to sophisticated conversion technology	Oil extraction techniques are costly	The initial expenditure and pilot setup are pricey
Water footprint	Potable water is required for the production of biofuel	Potable water is required for the production of biofuel	Can use sewage, salty, and nonpotable water to generate biofuel	Can use sewage, salty, and nonpotable water to generate biofuel
Nutrient requirements	Dependent on fertilizers and insecticides	Not depending on any fertilizer’s treatment	Dependent mostly on carbon and nitrogen supplies. Nutrients may be recycled, and solar energy**s**can be utilized	Dependent mostly on carbon and nitrogen supplies. Nutrients may be recycled, and solar energy can be utilized
Chemical fertilizers and pesticides	Primarily utilized	Not consumed	Not consumed	Not consumed
Environmental risks	Pesticides and fertilizers pose a hazard to the environment	The major concern is deforestation	The major concern is marine eutrophication	GMO release in environment can be threat
Harvesting	Picking can be accomplished using either mechanized or manual ways	Done by hand or machine picking	Microalgae harvesting is complicated and financially expensive	Microalgae harvesting is complex and financially costly

### Classification of biofuels according to their physical state

Biofuels are roughly categorized into three major groups depending on their physical condition at room temperature:

#### Solid biofuels

Solid biofuels, derived from non-fossil organic materials, such as plant biomass, animal waste, and municipal waste, are used for heating, cooking, and electricity generation. These plants have evolved from wood since fire discovery to biochar, forest products, and other renewable sources [112].

#### Liquid biofuels

Liquid biofuels encompass all fuels derived from natural biomass or biodegradable components. Compared to solid/gaseous biofuels, liquid biofuels such as bioethanol, biodiesel and bio-oil have higher energy density, benefiting transportation, storage and retrofitting [58]. These materials can be categorized as: triglyceride-based biofuels, including vegetable oils, pyrolytic oils, biodiesels and biogasoline, and or as lignocellulosic-based biofuels, such as bio-oils and biomass-to-liquid diesel (BTL) [73, 108]. In use for more than two decades globally, liquid biofuels are typically blended with petrol and diesel for retail. Major consumers include Australia, the US, Canada, Europe, Asia and South America, utilizing substantial volumes of the country [102]. In summary, easy-to-handle liquid biofuels produced sustainably from biomass and waste can be used to directly replace or blend with fossil fuel equivalents.

#### Gaseous biofuels

Biomass pyrolysis and redox reactions produce gaseous biofuels, including biogas, biohydrogen, and bio syngas, which are low-density, renewable biomass energy sources [125, 141]. Biowastes are converted into gaseous biofuels through pyrolysis or gasification, which are used in Otto engines for electricity generation. Biomass energy is being developed to reduce fossil fuel use. Two methods are used: combustible gas production by incomplete combustion and pyrolysis of biomass, and biogas production by anaerobic bacteria [125].

## Triglyceride conversion technology

Triglycerides (TG) are often found in animal fats and plant oils and can be utilized to produce biofuels. Triglycerides may be converted into biofuels via transesterification, thermal cracking, or hydrogenation methods. Table 3 summarizes the areas of potential for enhanced methods used to produce biofuels from TG-based biomass [51].

**Table 3:** Overview of the possibilities of advanced technology in the manufacture of triglyceride-based biofuels [99]

Technology	Biofuel	Areas of opportunity
Static mixers	Biodiesel	There is a need for reliable design and scaling methodologies. It is critical to guarantee that the technology's advantages are maximized when scaling
Sono-reactor	Biodiesel	There is a need to establish design techniques for industrial-scale deployment In industrial-scale systems, energy distribution must be uniform
Microwave reactor	Biodiesel	There is a need to establish design techniques for industrial-scale deployment. It is required to create appropriate technologies to produce microwaves on an industrial scale
Oscillatory flow reactor	Biodiesel	There is a need to provide trustworthy design and scalability techniques. It is critical to guarantee that the technology's benefits prevail while scaling
Microreactor	Biodiesel	There is a need to provide trustworthy design and scalability techniques. It is vital to assess the viability of deploying parallel arrangements on an industrial scale. It may be enough for analyzing the viability of creating comparable systems on a larger scale
Supercritical technologies	Biodiesel	More exact thermodynamic, transport and kinetic models are needed to examine the performance of supercritical systems. It is critical to develop ways for reducing the energy usage of these sorts of procedures. Strategies for minimizing the required reaction conditions and excess alcohol must be devised
Membrane reactor	Biodiesel	Low-cost membranes with superior mechanical and thermal resistance must be developed. Technologies for preventing membrane fouling must be developed
Bifunctional catalysts	Biojet fuel	It is critical to develop catalysts that enable the performance of processes with lower hydrogen requirements
Thermally coupled distillation	Biojet fuel	Alternative intensification technologies must be developed to further reduce the energy requirements for the various components and compositions utilized in biojet fuel production
Reactive distillation	Biodiesel/biojet fuel	There is a need to develop low-cost catalysts appropriate for industrial use. Waste-based catalysts should be tested for biofuel production in reactive distillation systems Strategies for decreasing energy requirements must be implemented to guarantee that the proposed systems have adequate control capabilities
Reactive extraction	Biodiesel	Adequate solvents must be developed to ensure high extraction efficiency and yields at a low cost Strategies to avoid catalyst deactivation must be developed
Reactive absorption	Biodiesel	

While biodiesel produced by transesterification is commonly mixed with petroleum diesel, its poor density and stability do not match the needs of modern transportation engines. Green diesel, manufactured from hydrocarbons, is created utilizing thermal cracking and hydrogenation processes [170].

Thermal cracking can change raw materials with high acid contents and undesirable impurities, including trash and acidic oils. The resulting hydrocarbon-based biofuel can then be combined with petroleum diesel at any ratio [48, 51].

Green diesel generated by upgrading procedures (e.g., aromatization and isomerization) has a lower cold-filter point, better economic value, and more diverse uses. Microalgal oil cultivation and culture technologies are now being explored in Europe, the United States, South Korea, Japan, China, and other regions. China also plans to employ woody oils such as those from Tung and Jatropha as raw sources for biofuels. Overall, the cultivation of triglyceride raw materials should comply with the premise of “not competing with present food crops and acreage for food production” [51, 170, 173].

To improve green diesel production efficiency,

- (i)The development of stable and active solid base catalysts for biodiesel requires chemical conversion and lipase immobilization to enhance enzyme activity, thermal stability, and methanol tolerance. This can be accomplished by genetic engineering, protein modification, or guided approaches [51].
- (ii)Advancements in production technologies have led to fuels that outperform green diesel in terms of combustion efficiency [48].
- (iii)Future research should aim to enhance the catalytic fundamentals and create low-cost, active, and stable catalysts for directed deoxygenation conversion [51, 170].
- (iv) Photocatalytic hydrogenation conversion, a new triglyceride conversion method that operates under mild reaction conditions, consumes little energy and hydrogen, and achieves high conversion and selectivity, is a hot topic of research. Research and development efforts are needed to find new catalysts [48, 87].

## Biomass conversion technologies

Biomass conversion techniques encompass physicochemical, thermochemical, and biochemical processes, and utilize physical, chemical, and biological methods to produce biofuels [18]. The choice of biomass–energy conversion method depends on factors such as energy density, size, moisture content, and intermittent supply. Biomass is recognized as a crucial renewable energy source that offers advantages such as long-term sustainability, lower environmental impact in terms of carbon dioxide (CO_2_) and sulfur emissions, and greater economic viability than fossil fuels [167]. Consequently, companies are increasingly adopting biomass conversion technology to produce biofuels for diverse energy applications. The global biomass supply has been steadily expanding, with solid biomass accounting for 85% and liquid biofuels experiencing a 2% annual growth rate from 2000 to 2018 [41, 106]. These technologies present opportunities for sustainable energy generation, reducing reliance on fossil fuels, and mitigating environmental impacts. Ongoing research and development efforts aim to enhance conversion efficiency, optimize product yields, and improve the economic and environmental sustainability of biomass conversion processes.

### Different methods of biomass conversion

**Fig. 12:**
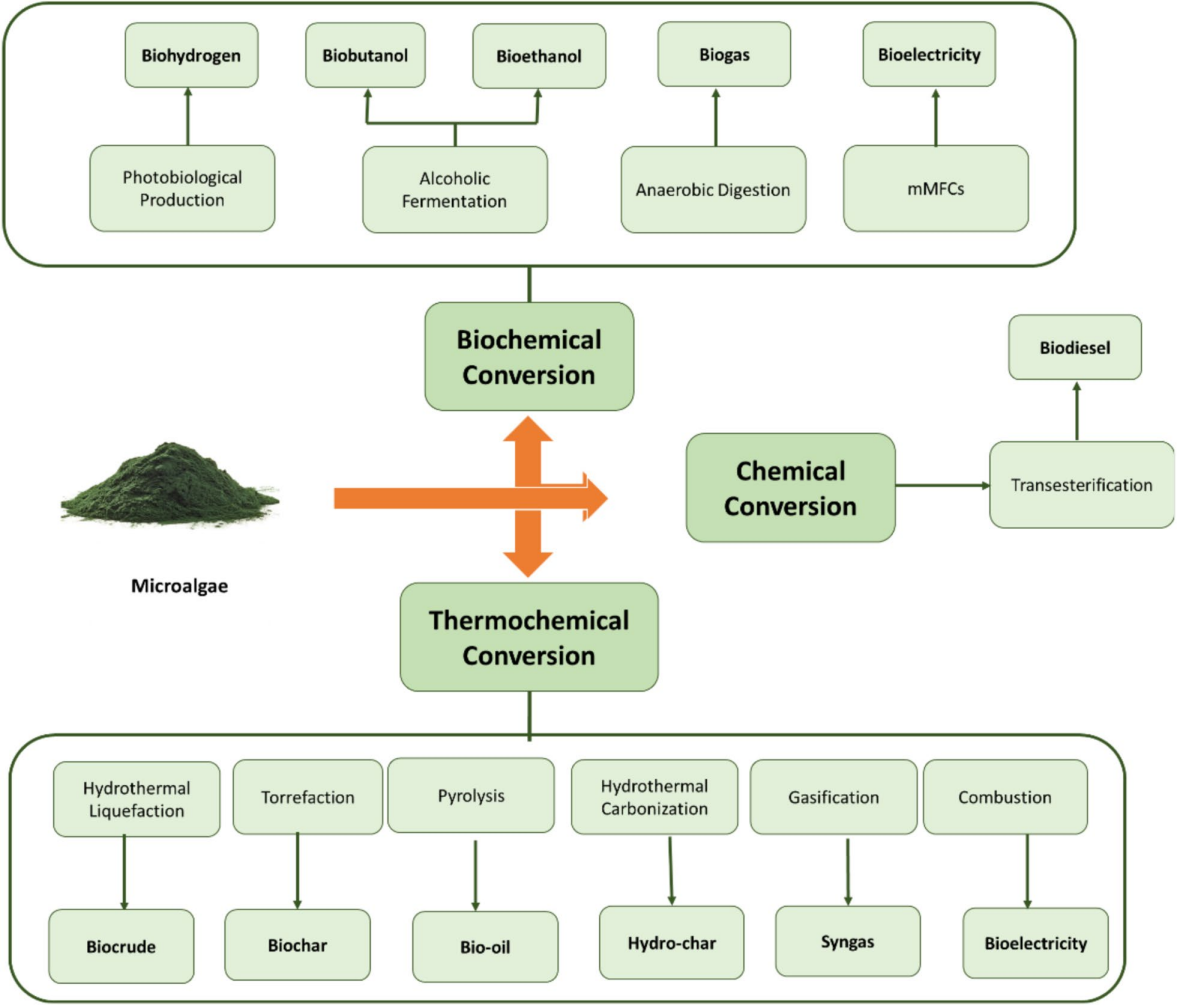
Different types of algal biofuels and conversion methods [8]

Biomass conversion to energy is accomplished through two key processes: thermochemical conversion and biochemical transformation. These technologies convert biomass into three important products: biofuels, heat, and power for electricity generation. As a specific example of biomass conversion, Fig. 12 illustrates various conversion methods and products for microalgal biomass, which represents an important subset of biomass feedstocks.

#### Thermo-chemical processes

The thermos-chemical conversion of biomass can be further characterized by high-pressure liquefaction and rapid pyrolysis. Biomass, primarily bio-oils derived by thermochemical conversion, plays a significant role in the fuel industry as an alternative source of fuel and chemicals [98].

##### Combustion

Is one of the earliest and most basic ways of biomass conversion, in which organic materials are burnt to generate heat and energy. Combustion is the process of burning biomass in the air and converting chemical energy into heat, mechanical power, or electricity. The combustion process is carried out using a range of process equipment, such as stoves, furnaces, boilers, steam turbines, and turbo-generators [42]. The primary combustion products are heat, electricity, or combined heat and power (CHP) from biomass combustion, which is an effective strategy that utilizes both heat and power through successive heterogeneous and homogeneous processes [23, 42]. The particle size of the feedstock, temperature, and combustion environment all have major impacts on biomass combustion. The fundamental constraint of the combustion process is the substantial emission of carbon dioxide and nitric oxide, as well as the discharge of particulates and ashes.

##### Pyrolysis

Is a thermal breakdown process that converts biomass into charcoal, bio-oil, and syngas, which is a key component in biofuel production. It transforms long-chain molecules into short-chain molecules, resulting in concentrated fuel oils [98]. Typically, biomass or waste is employed as the primary feedstock for producing syngas and other liquid fuels by varying the process parameters [4, 140]. The primary advantages are the conversion of solid materials into gases and vapors that are easy to handle, transport, and store. However, the disadvantages of these methods include the high heat input needed to carry out the chemical reactions for the formation of syngas. [27, 138].

##### Gasification

Gasification is a thermochemical process that heats biomass under oxygen-limited circumstances, producing syngas, a mixture of carbon monoxide and hydrogen that may be used to create power, heat, or biofuels [17]. Combined cycle plant systems are similar to gasification conversion systems. It is widely used due to the availability of clean gas before combustion in turbines. Cleaning and compacting gas greatly reduces its volume [23, 151].

##### Liquefaction

This method converts biomass into liquid fuels using heat and a catalyst, producing bio-oils and biofuels from various feedstocks. It involves low temperatures and high hydrogen pressure, producing a complex mixture of volatile organic acids and hydrocarbons [17]. Catalytic liquefaction, assisted by a catalyst or high hydrogen partial pressure, effectively generates products with greater energy density in the liquid phase [106, 111].

##### Hydrothermal processing

Is a thermochemical conversion method that utilizes high-temperature and high-pressure water to convert wet biomass into biofuels or biochemicals. This technique is applicable to a wide range of feedstocks, including algae and waste materials. The process occurs in an aqueous environment at subcritical and supercritical temperatures (180 to 375 °C) and pressures (2 to 22 MPa). Under these conditions, biomass components undergo degradation in water, resulting in the production of biofuels such as hydro char, biocrude, and syngas through hydrothermal carbonization (HTC), hydrothermal liquefaction (HTL), and hydrothermal gasification (HTG), respectively.

Hydrothermal processing techniques operate under different temperature and pressure conditions. Hydrothermal carbonization (HTC) typically occurs at temperatures between 180 and 260 °C and pressures of 2–6 MPa, resulting in the production of a coal-like solid called hydrochar [95]. Hydrothermal liquefaction (HTL) operates at temperatures ranging from 280 to 370 °C and pressures of 10–25 MPa, primarily yielding a liquid bio-crude oil product [50]. Hydrothermal gasification (HTG) takes place at higher temperatures, typically 400–700 °C, and pressures above 25 MPa, converting biomass primarily into gases such as hydrogen and methane [169]. These varying conditions allow for the targeted production of different biofuel products from biomass feedstocks.

The specific process parameters and conversion pathways are determined based on the desired product. Hydrothermal processing offers a promising approach for effectively converting lignocellulosic biomass into energy and fuels, addressing the challenge of accessing biomass resources that are abundant in nature but often contain high moisture contents. To advance the understanding of hydrothermal conversion, it is crucial to investigate biomass degradation under hydrothermal conditions and the chemistry of product formation [17, 107].

#### Biochemical processes

Biochemical (or biological) conversion technologies utilize microorganisms to convert biomass into economically viable gaseous products with a wide range of uses [91]. Fermentation and anaerobic digestion are the two primary methods employed.

##### Fermentation

Fermentation is a commercial method that produces bioethanol from sugar- and starch-rich biomass such as maize and sugarcane. This process entails breaking down saccharides, turning sugars to ethanol, and purifying the byproducts. Sugarcane is an ideal feedstock because of its high productivity and energy potential [52, 91, 111, 155].

##### Anaerobic digestion

Anaerobic digestion is a process in which bacteria break down organic matter without oxygen, producing methane-rich biogas. This energy-efficient and eco-friendly technology is used for electricity generation and fuel production. The digestate is used as fertilizer and biogas as fuel, while biosolids are used for soil conditioning [2, 7, 31].

## Biofuel examples

Biofuels are widely seen as a cost-effective and ecologically friendly alternative to petroleum and other fossil fuels, especially in light of rising petroleum prices and growing concern about the impacts of fossil fuels on global warming. Below are some examples of biofuels:

### Bioethanol

Yeast cells are the principal source of ethanol production, digesting carbohydrates in the absence of CO_2_. The fermentation of sugar and starch yields bioethanol, which is a sustainable energy source.

Bioethanol is a first-generation biofuel derived from agricultural products such as maize, sugarcane, potatoes, and rice [106]. In addition to these traditional feedstocks, bioethanol can also be produced through the fermentation of carbon monoxide using specialized bacteria. This process, known as gas fermentation, utilizes anaerobic bacteria such as Clostridium autoethanogenum to convert carbon monoxide into ethanol [96]. This approach offers the potential to produce bioethanol from industrial waste gases, thereby reducing carbon emissions and providing an additional pathway for sustainable fuel production [109]. Ethanol costs approximately one-third less than petrol. One liter of ethanol is comparable to approximately 0.65 L of gasoline. Ethanol has a larger energy content than petrol, with 11.3 MJ/liter. Ethanol also has a lower vapor pressure, making it easier to store in pure fuel form than petrol [27]. The EU quality standard specifies a 5% blend of bioethanol and petrol. This blend does not require any engine adjustments and may be used at higher levels. The United States (US) generates the most ethanol (10% ethanol, 90% petrol), followed by Brazil (27% ethanol) [1, 85].

Due to energy scarcity and environmental concerns, bioethanol, a possible biofuel source, has been investigated utilizing a variety of biomass resources, such as sugar products, starch, and lignocellulosic biomass [104].

**Fig. 13:**
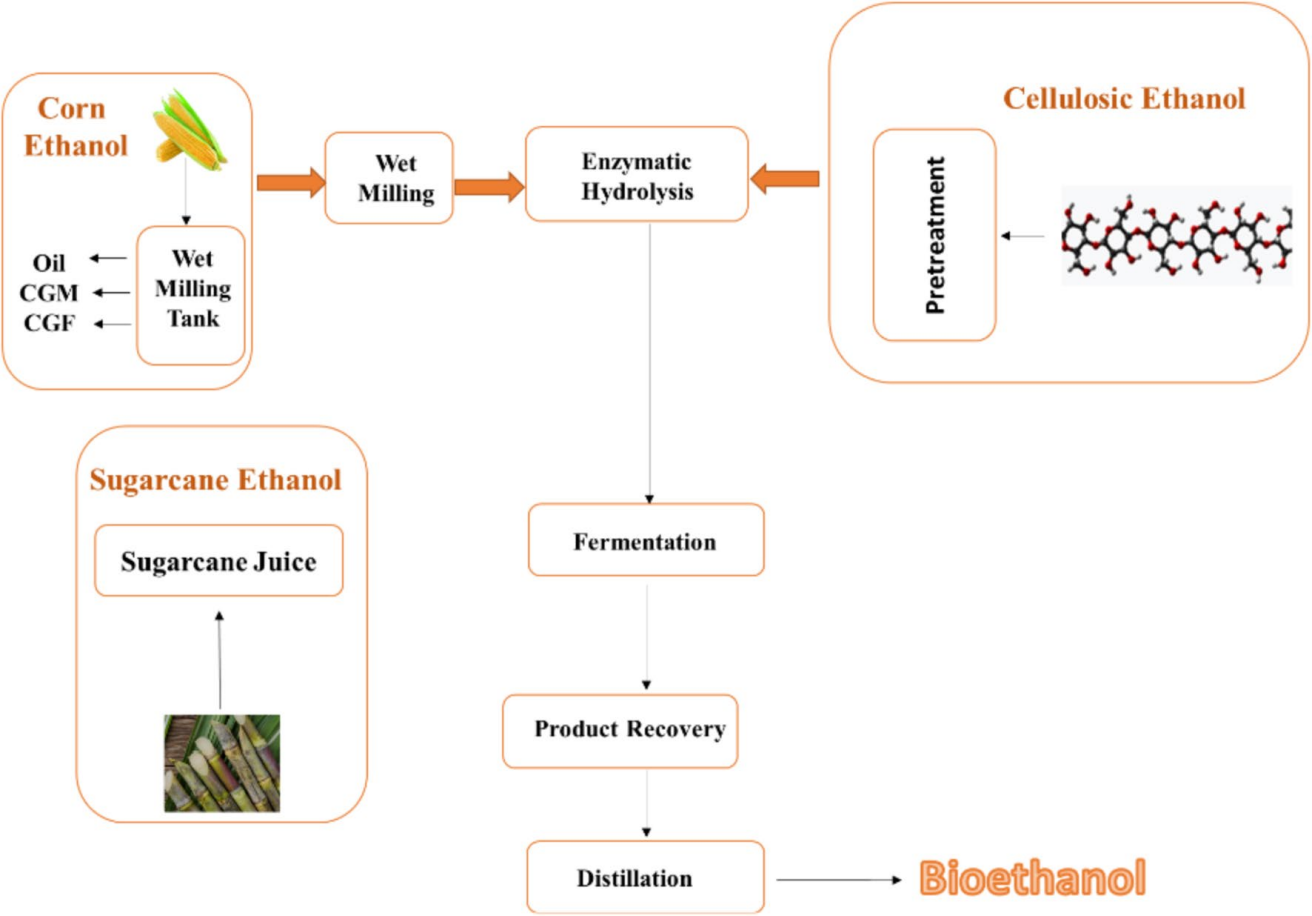
Production of bioethanol from three commonly used sources: corn, sugarcane, and cellulosic biomass [149]

Bioethanol is produced from sugar-containing materials such as sugarcane, corn, and algae through a three-step process: preparation, fermentation, and recovery and concentration (Fig. 13) [149]. Recent advancements in second-generation bioethanol production from lignocellulosic biomass emphasize meticulous monitoring of each stage, including pretreatment, enzymatic hydrolysis, fermentation, and distillation. Despite its safety, environmental friendliness, and low energy consumption, biological pretreatment remains a promising biomass resource [104]. Table 4 summarizes the biofuel yield of the second-generation biomass.

**Table 4:** Bioethanol production from second-generation biomass

Biomass feedstock	Bioethanol production (L/kg dried biomass)
Corn stover	5.87
Rice straw	116.75
Barley straw	0.053
corn meal	9.77 ± 0. 12
Palm oil residue	110 600–172 100
Paddy straw	0. 00086
Eucalyptus	0.614_S_
Olive tree pruning	0. 023
Palm wood	0. 0228

### Biodiesel

Biodiesel is produced from triglyceride oil, derived from vegetable oil, animal fat, or microalgae-derived triglycerides, with algae-producing oils in high demand, while macroalgae have a low oil content [20]. A triglyceride molecule has three fatty acid moieties linked to a glycerol residue [48]. Transesterification is the process of converting triglycerides into fatty esters and glycerol, including biodiesel, using commercial catalysts such as sodium hydroxide or potassium hydroxide, with alkoxides becoming more effective [20, 41].

**Fig. 14:**
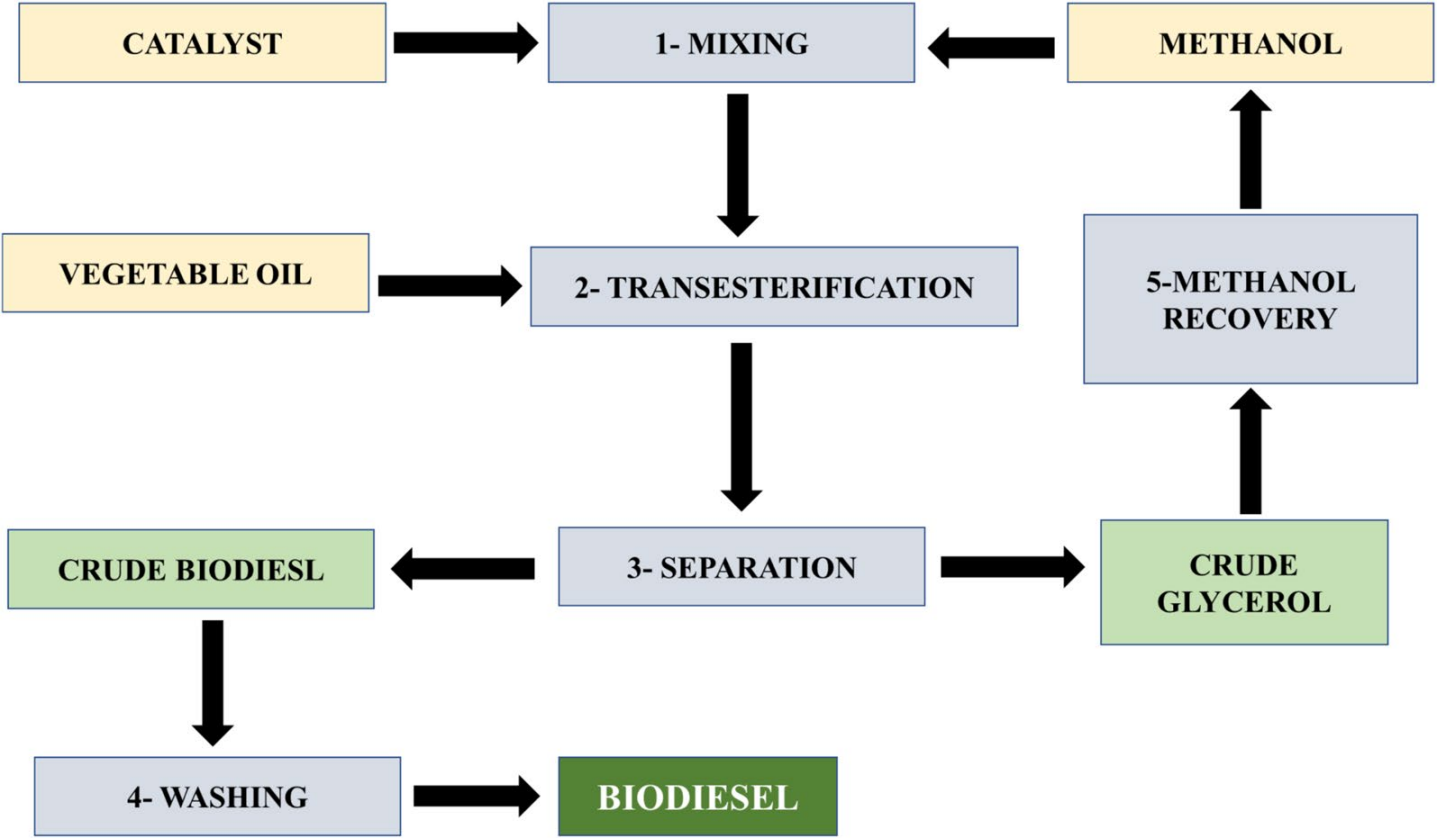
Steps involved in biodiesel production [144]

The alkali-catalyzed transesterification process occurs at approximately 60 °C and atmospheric pressure. The reaction normally takes approximately 90 min and yields more than 98% of the potential yield. The reaction consisted of two phases: methanol and oil. Following this method, the oil phase was biodiesel, while the oil-unmixable phase was composed of glycerol and residual methanol. The biodiesel phase is collected and thoroughly washed with water to eliminate any leftover methanol, glycerol, or catalyst. Figure 14 illustrates the processes involved in producing biodiesel from vegetable oil [144].

### Biomethanol

**Fig. 15:**
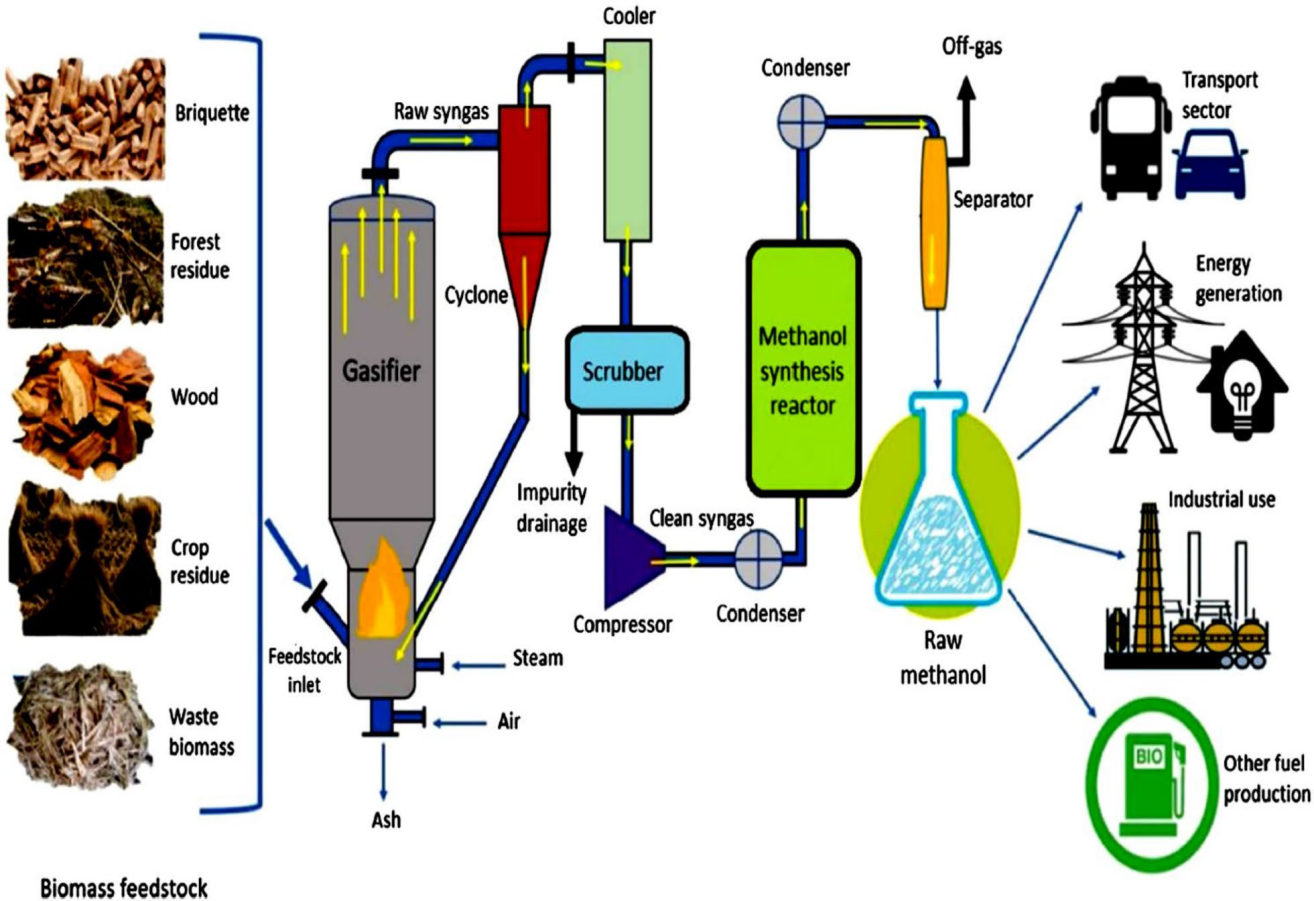
Production of biomethanol by gasification [3]

Biomethanol is another possible biofuel that might replace traditional motor fuels. Methane is the simplest hydrocarbon, with the general formula CH4, and it occurs in gaseous form at normal temperatures and pressures [13]. Previously, it was utilized for power autos, and a synthetic version was widely used in Germany during WWII. Methanol, often known as 'wood al-cool’, is now manufactured from natural gas via partial oxidation, whereas bioethanol requires the distillation of liquid resulting from wood pyrolysis. Biomethane consists of several purification techniques, the bulk of which include methane (> 80% concentration) and CO_2_ [97]. Biomethanol production using high-temperature conversion of RDF (refuse-derived fuel) is a highly promising approach. Improvements in high-temperature conversion and other production lines synchronize this section’s on-stream time with the rest of the plant, resulting in approximately 8400 h of operation per year [64]. BiomBio-methanol production using high-temperature conversion of RDF (refuse-derived fuel) is a highly promising approach. Improvements in high-temperature conversion and other manufacturing lines synchronize this section’s on-stream time with the rest of the plant, yielding approximately 8400 h of operation per year [92–94]. A biomethanol-generating facility has properties that are almost identical to those of coal gasification-based methanol production. Fossil-based methanol facilities commonly employ the following procedures: (a) gasification, (b) gas cleaning, (c) hydrocarbon reforming, (d) water–gas shift reaction, and (e) hydrogen addition and/or CO_2_ removal, and (f) methanol synthesis and filtering [30, 92–94]. Figure 15 depicts a gasification-based methanol generation process that involves producing raw syngas from various biomass feedstocks; passing it through cyclone, cooler, and scrubber units; and synthesizing it in a reactor for use in transportation, energy generation, and industrial applications. Pretreatment of raw materials is required for biomethanol synthesis, which is predominantly derived from biomass sources. The treated biomass is then gasified into syngas. To optimize hydrogen and carbon monoxide synthesis while reducing undesired water and carbon dioxide amounts, oxygen delivery is controlled during feedstock heating above 700 °C. Contaminants and impurities are removed before the product gas undergoes multiple conditioning procedures to optimize its composition. Syngas conditioning is primarily used to generate syngas that contains at least twice as many hydrogen molecules as carbon monoxide molecules. The nature of the initial syngas and the availability of hydrogen determine the optimal hydrogen-to-carbon monoxide (H_2_/CO) ratio. There are numerous methods for adjusting the levels of hydrogen and carbon monoxide [65, 129]. The main agricultural remnants or wastes and their CH_4_ production are shown in Table 5 [114, 159].

**Table 5:** quantifying methane production from the anaerobic digestion of agricultural residues [114]

Agricultural residues	CH_4_ yields (mL/g volatile solids) by anaerobic digestions
Fruits and vegetable wastes	180–732
Wheat straw	290
Rice straw	302
Oil palm fruit bunch	276–340
Spent coffee grain	296
Riso stillage	324
Swedish stillage	485
Corn stover	338
Wheat stillage	380–529
Cattle manure	398
Pig manure	495
Orange peel	217
Maize silage	236
Grass silage	361
Raw pig slurry	330

### Bio-oil

Bio-oil is a liquid product produced by the pyrolysis of biomass, and is a thermochemical conversion process that includes heating biomass in the absence of oxygen. Bio-oils are complex combinations of organic chemicals, such as phenols, ketones, esters, alcohols, and hydrocarbons, as well as water and contaminants. It is dark brown in color and contains a high oxygen concentration, making it chemically unstable and unsuitable for direct use as fuel. However, bio-oil may be improved by different procedures to remove contaminants and lower the oxygen concentration, making it a desirable feedstock for the manufacture of fuels, chemicals, and other high-value goods [54]. These upgrading procedures can be broadly categorized into physical, chemical, and catalytic methods:

#### Physical methods

- Filtration: removes solid particles and char [168].

- Solvent addition: reduces viscosity and improves stability [10].

#### Chemical methods

- Emulsification: forms a stable micro-emulsion with diesel fuel [28].

- Esterification: reduces acidity and improves stability [122].

#### Catalytic methods

- Hydrodeoxygenation (HDO): removes oxygen through catalytic reaction with hydrogen, producing water and saturated carbon–carbon bonds.

- Catalytic cracking: breaks down larger molecules and removes oxygen in the form of CO_2_, CO, and H_2_O [90].

- Hydrocracking: combines cracking and hydrogenation to remove oxygen and reduce molecular weight [79].

These upgrading processes aim to address the main challenges of raw bio-oil, including high oxygen content (typically 35–40 wt.%), high water content (15–30 wt.%), high acidity (pH 2–3), and chemical instability. By reducing oxygen content and removing contaminants, these methods improve the heating value, stability, and compatibility of bio-oil with conventional refinery processes and end-use applications.

The composition of biomass plays a crucial role in determining the yield and quality of bio-oil during pyrolysis, with different components of biomass influencing pyrolysis reactions and the resulting product distribution [54, 61].

**Fig. 16:**
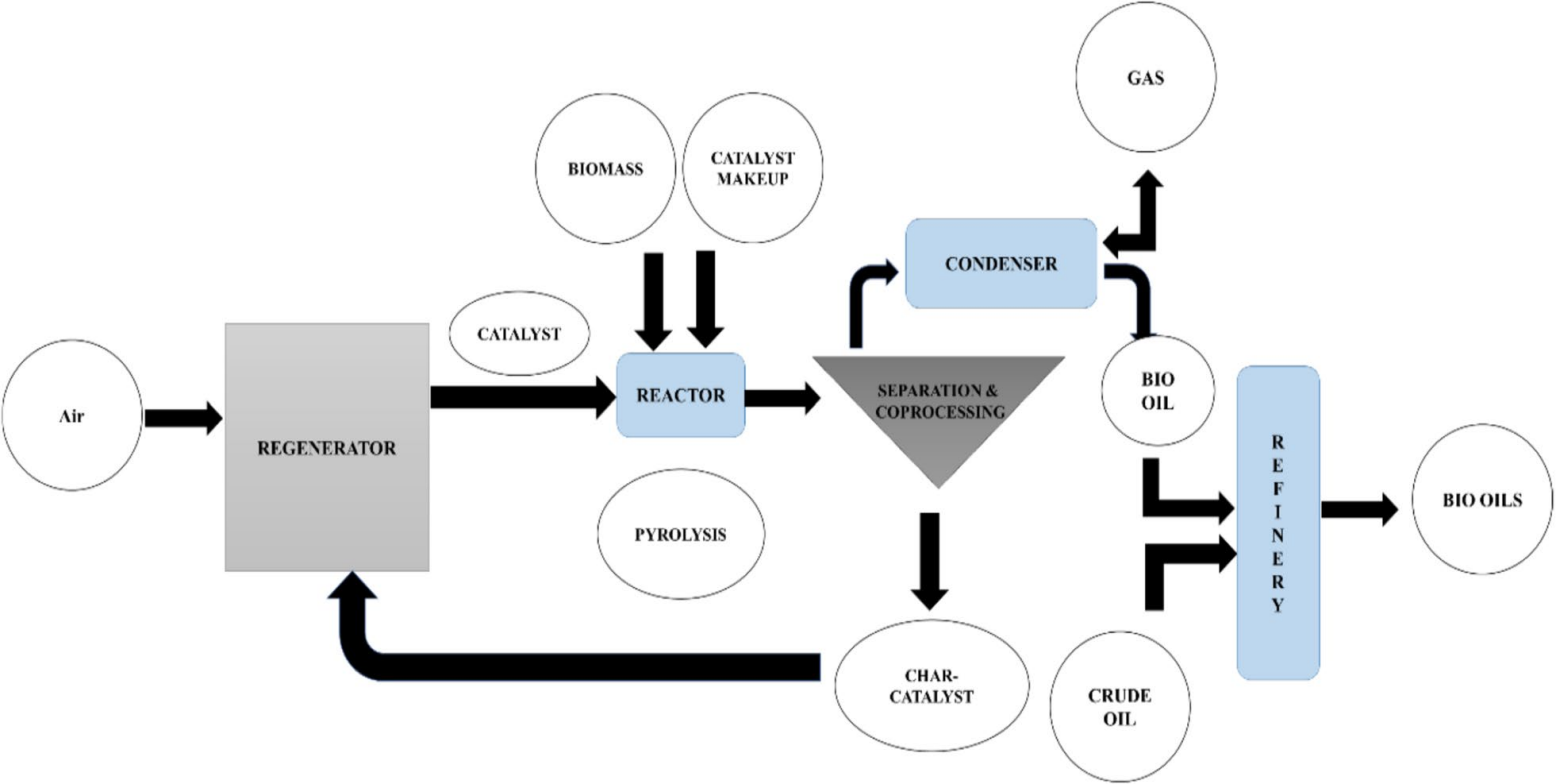
A schematic diagram of the production of bio-oil using the pyrolysis process

Bio-oil is produced through the pyrolysis of biomass, a thermochemical conversion process that involves heating biomass in the absence of oxygen; Fig. 16. The resulting bio-oil has a high oxygen content, making it unstable and initially unsuitable for direct fuel use. However, various upgrading processes can be applied to improve its quality and suitability as a fuel or feedstock for other high-value products [57, 171].

### Biohydrogen

**Fig. 17:**
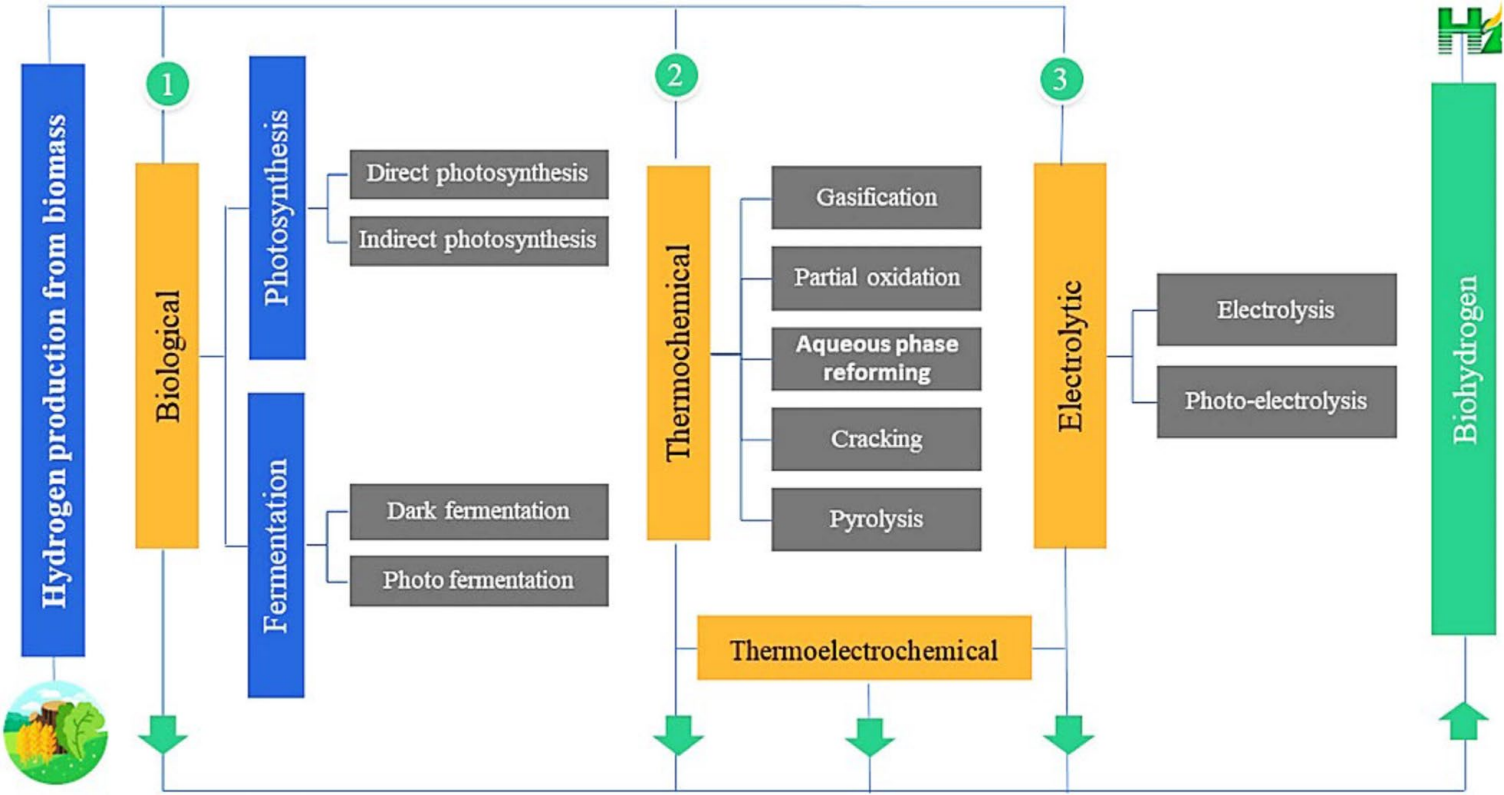
Main pathways for H_2_ production based on biomass [40]

Biohydrogen is a clean fuel created biologically from many types of biomass, including biological waste. Hydrogen is recognized as one of the most competent alternative fuels for generating future net-zero emissions. The advent of the envisioned biomass-to-sustainable H2 approach is considered an attractive way to produce a sustainable strategic H2 [174]. It is created by live microorganisms that transform hydrogen into chemical energy through photolysis or fermentation [123]. Biohydrogen production is interesting because it involves the use of clean fuel that can be easily created from certain types of biomass [40, 110]. Currently, the most prevalent methods for creating hydrogen from biomass are thermochemical, biological, and electrolytic processes (Fig. 17).

Biohydrogen yield is enhanced by the addition of catalysts in production processes such as bio-photolysis and fermentation. Biocatalysts, such as hydrogenases and nitrogenases, accelerate reaction rates without altering fermentation. Organic matter, enzymes, and metals also catalyze biohydrogen production, as microbes are sensitive to these substances [40, 124]. The agro-industrial waste feedstocks employed for biohydrogen production are given in Table 6.

**Table 6:** Assessing biohydrogen yield from anaerobic digestion of different agricultural waste feedstocks and processing methods [40]

Methods employed for biohydrogen production	Agricultural waste feeds stock	Maximum biohydrogen yield
Integrated biohydrogen process of dark fermentation and microbial electrolysis	Sugar beet juice	6 mol H_2_/mol h exose added
Sequential dark and photo fermentation	Barley	0.58 mmol/L/h
Anaerobic fermentation	Date seeds	1.330 mmol/L/h
	Rice straw	19.73 mL/g
	Sugarcane bagasse	6980 mL H_2_/L
	Apple pomace	134.04 mL H_2_/L
	Wheat waste	1.22 mol H_2_/mol glucose
	Potato waste	298.11 mL H_2_/g
	Cashew apple bagasse	12.57 mL H_2_/L/h
	Wheat straw	1–68 mL H_2_/g
Dark fermentation	Corn stoker	49–68 mL H_2_/g
	Cattle manure	65 mL H_2_/g VS 545 mL H_2_/L
	Rice bran	
	Rice bran deoiled cake	295 mL H_2_/L
	Pig manure	14–18 mL H_2_/g

### Biojet

Biojet fuel, commonly known as aviation biofuel, is a form of renewable fuel developed particularly for use in jet engines. It is made from sustainable and renewable biomass sources such plant oils, animal fats, algae, and waste products. Biojet fuel attempts to lessen the aviation industry’s dependency on traditional fossil fuels while also mitigating its environmental effect by providing a more sustainable alternative. The manufacture and use of biojet fuel have the potential to reduce greenhouse gas emissions and the industry's total carbon footprint [34, 166]. The aviation industry aims to produce biojet fuel from renewable biomass to help meet its emission-reduction goals. Currently, aviation accounts for 2% (705 million tons) of global CO2 emissions, but this value could increase to more than 340% to 3.1 billion tons by 2050 if conventional jet fuel remains dominant [34].

Sustainable aviation fuels (SAF) represent a crucial pathway for reducing the carbon footprint of the aviation industry. SAF can be produced from a wide range of non-petroleum-based renewable feedstocks, offering significant potential for greenhouse gas emissions reduction compared to conventional jet fuel. The feedstocks for SAF production include, but are not limited to:

- 1. Biomass: including agricultural residues, forestry residues, and energy crops [5].
- 2. Municipal solid waste (MSW): particularly the food and yard waste portions [25].
- 3. Fats, oils, and greases: including used cooking oils, animal fats, and tall oil (byproduct of wood pulp manufacture) [48].
- 4. Industrial off-gases: such as waste carbon monoxide from steel mills [24].
- 5. Algae: both micro and macroalgae are being explored for their potential in SAF production [6].
- 6. Atmospheric CO2: direct air capture (DAC) technology combined with renewable hydrogen can produce synthetic SAF [146].

Each feedstock presents unique advantages and challenges in terms of availability, conversion efficiency, and overall sustainability. For instance, waste-derived feedstocks like MSW and used cooking oils offer the benefit of recycling waste streams, while purpose-grown crops raise concerns about land use change and competition with food production [64].

The choice of feedstock significantly influences the production pathway and the final fuel characteristics. Currently, the most common pathways for SAF production include hydroprocessed esters and fatty acids (HEFA), Fischer–Tropsch (FT) synthesis, alcohol-to-jet (ATJ), and synthesized iso-paraffins (SIP) [161].

Ongoing research is focused on improving conversion efficiencies, reducing production costs, and developing new pathways to utilize a broader range of feedstocks, with the ultimate goal of increasing SAF production to meet the growing demand for low-carbon aviation fuels.

There are major public and private research initiatives investigating economical and eco-friendly methods for manufacturing biojet fuel at scale to replace petroleum-based jet fuel. Multiple renewable biomass sources and conversion processes are being explored to produce viable biojet fuel alternatives [55]. Biojet fuel may be created from a number of renewable biomass sources utilizing various conversion processes. Some of the most prevalent manufacturing paths for biojet fuel are include the following.

#### Oil-to-jet (OTJ)

**Fig. 18:**
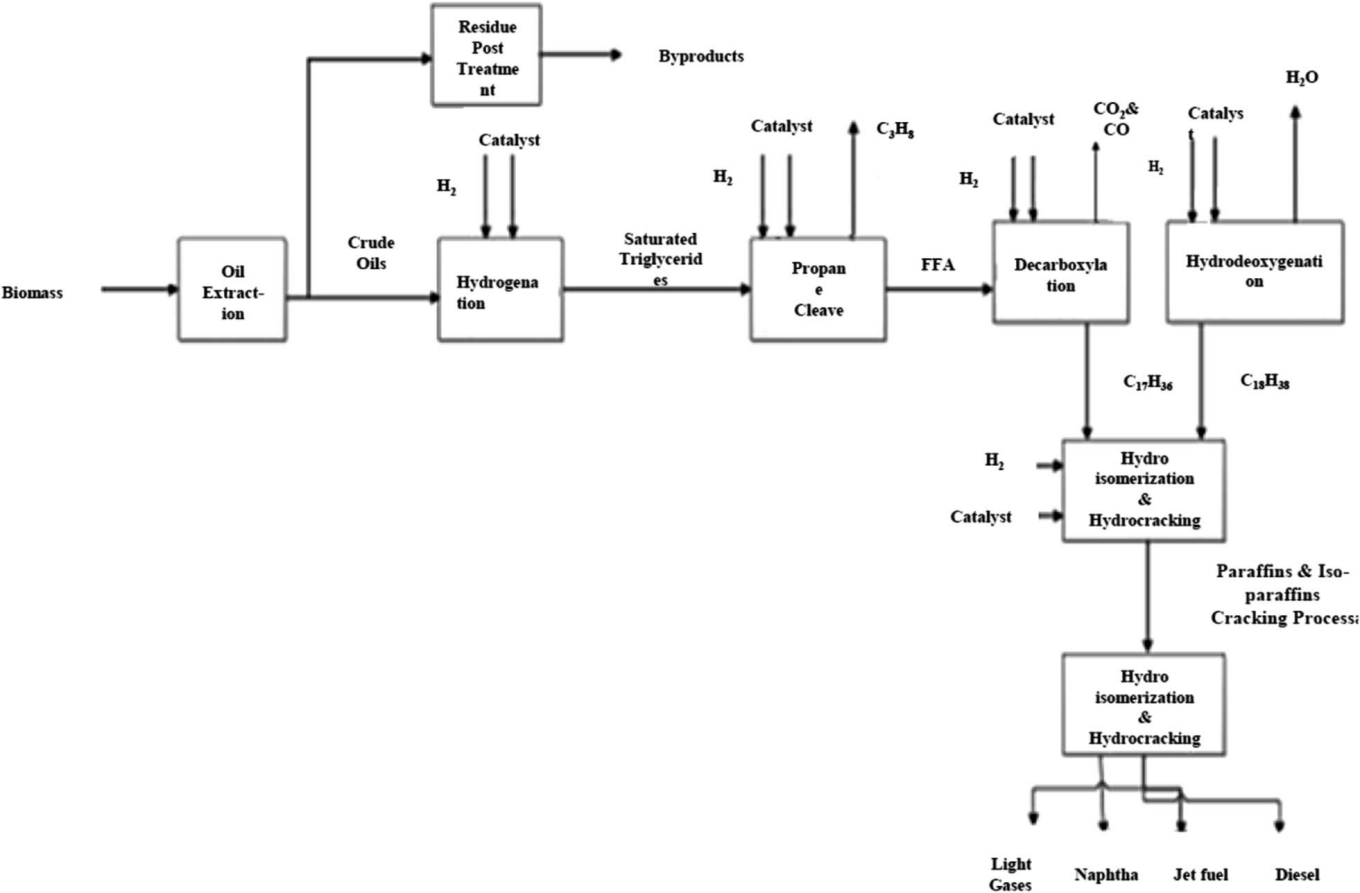
Hydroprocessed renewable Jet (HRJ) process

Conversion is a process in which plant or animal oils and lipids are transformed into straight-chain alkanes. There are four major methods for producing jet fuel from oils: hydroprocessing, catalytic hydro thermolysis, hydroprocessed hydrocarbons, esters, and fatty acids synthetic paraffinic kerosene, and hydroprocessed depolymerized cellulosic jet (pyrolysis) [45, 166]. A representative process flow diagram is shown in Fig. 18.

#### Sugar-to-jet (STJ)

Sugars are transformed into hydrocarbon fuels via dehydration, oligomerization, distillation, and hydrogenation processes. Direct fermentation of carbohydrates into hydrocarbons is also employed as a manufacturing route [55].

#### Gas-to-jet (GTJ)

It is a process that converts biomass to liquids by gasification/Fischer–Tropsch (FT) synthesis. Syngas generation, gas purification, FT synthesis, and product upgrading are the four fundamental processes of FT product production [161].

#### Alcohol-to-jet (ATJ)

This technique converts an alcohol (ethanol or butanol) intermediate derived from biomass into jet fuel. In addition to these methods, coprocessing of lipids and unprocessed hydrocarbons in an FT reactor with a medium distillate of crude oil in petroleum refineries has been permitted for the generation of biojet fuel [21, 55].

## Advances in biofuel production

Biofuels are an essential component of the renewable energy mix, and have the potential to reduce greenhouse gas emissions and reliance on fossil fuels [70]. Concerns about global oil price volatility, energy supply security, global warming, and the creation of new agricultural prospects are among the primary motivating causes for biofuel research. Furthermore, ambitious government regulations, energy security, concerns about sustainable agriculture, and a decrease in CO_2_ emissions in the transportation sector have all become key drivers of biofuel production growth. Biofuel production growth’ refers to:

- 1. Increased volume: a rise in the total amount of biofuels produced annually on a global scale.
- 2. Expanded capacity: the development of new production facilities and the expansion of existing ones.
- 3. Technological improvements: advancements in production processes that increase efficiency and yield.
- 4. Diversification: the production of a wider range of biofuels from various feedstocks.
- 5. Geographic expansion: the spread of biofuel production to new regions and countries.

This multifaceted growth in biofuel production contributes to the increasing use of biofuels as alternatives to fossil fuels [12]. It is important to note that this growth varies across different types of biofuels and geographic regions, and is subject to ongoing debates about sustainability and competition with food production.

IRENA’s low-carbon road to 2050 proposes a fivefold increase in biofuel usage, from 130 billion liter in 2016 to approximately 650 billion liters by 2050. This underscores the significance of ongoing research and development in the field of biofuels to fulfill the rising demand for sustainable energy [70].

Recent improvements in biofuel technology have focused on improving the efficiency of production processes, lowering production costs, and expanding the spectrum of feedstocks that may be utilized to make biofuels [75]. Cocultivation systems have been created to increase the efficiency of biofuel production, by employing different microorganisms to generate biofuels, chemicals, and other products [5]. However, there are still issues connected with coco-cultivation systems that must be solved to fulfill future industrial demands [119].

**Fig. 19:**
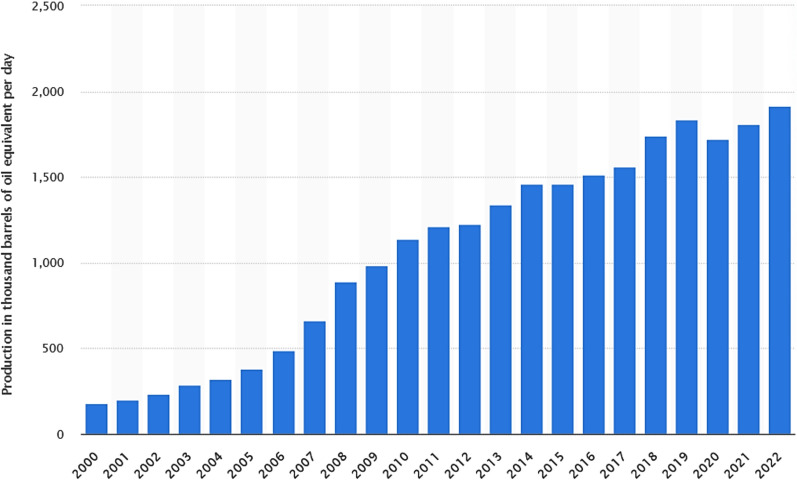
Biofuel production worldwide from 2000 to 2022 [5]

In 2022, global biofuel production reached 1914 thousand barrels of oil equivalent per day, a substantial increase from the 180 thousand barrels of oil equivalent per day produced in 2000 (Fig. 19). Regulations promoting the use and production of biofuels have largely fueled growth, with the assumption that they will enhance energy security and reduce greenhouse gas emissions in vital industries. Blending restrictions, environmental goals, fuel quality requirements, and import levies all have an impact on the biofuel industry. The global biofuel sector is expected to be worth more than $200 billion by 2030 [5].

Here are further details on the recent advances and developments in biofuel production:

### Advanced first-generation biofuels

Advances in sugar and starch-based ethanol production have improved efficiency and yield through genome shuffling and global transcription machinery engineering, leading to increased production rates and improved stress tolerance [135].

Research has aimed to expand the use of biodiesel feedstocks, including vegetable oils, animal fats, and cooking oils, to promote diverse feedstocks, reduce competition with food production, and enhance sustainability [135, 136].

### Advanced second-generation biofuels

Research on cellulosic ethanol, produced from lignocellulosic biomass has led to advancements in efficient pretreatment methods, such as steam explosion, acid hydrolysis, and enzymatic hydrolysis, and the development of robust enzymes [134, 148].

**Fig. 20:**
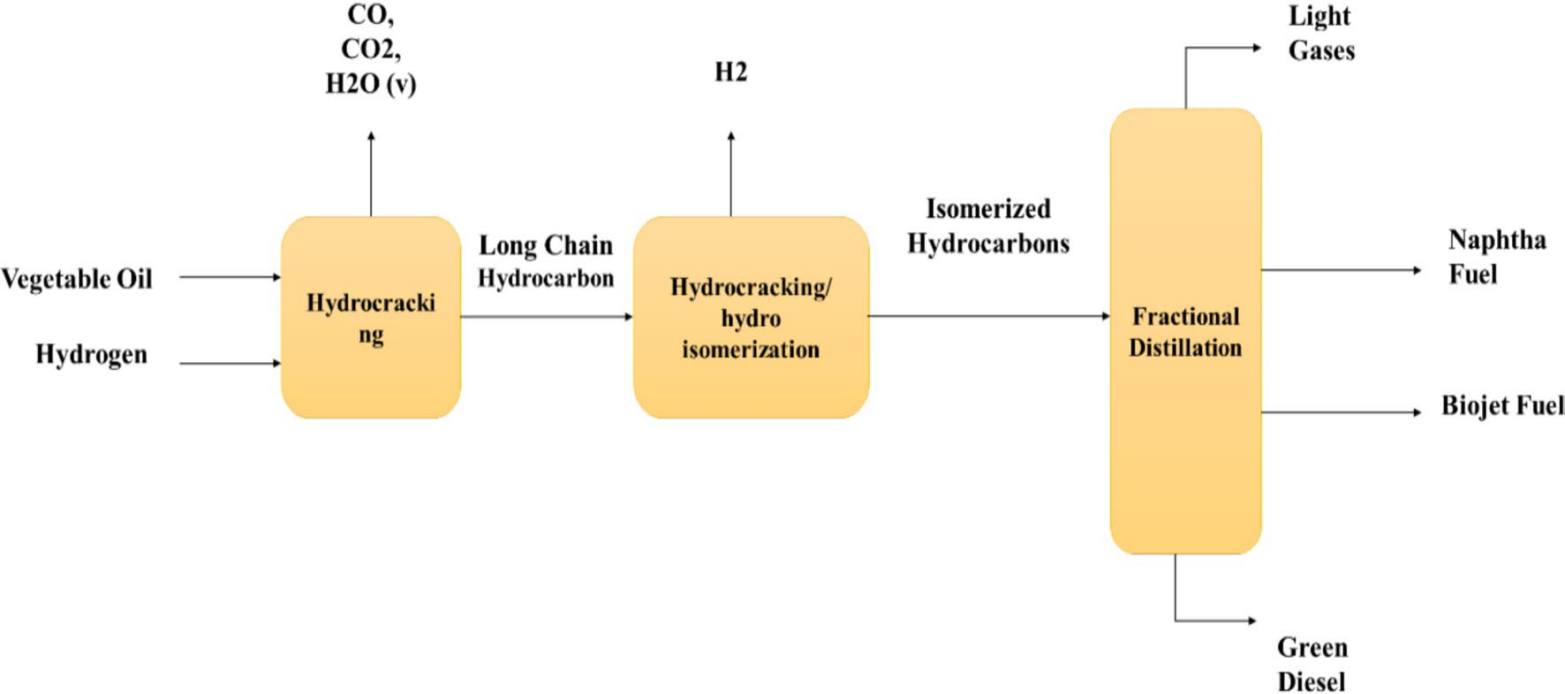
Diagram of the hydrothermal treatment process

- Other second-generation biofuels: This study revealed that hydrotreated vegetable oil (HVO) is an advanced biofuel made from animal fat and plant oil. The treatment of vegetable oil involves the introduction of hydrogen molecules into the raw fat or oil molecules. This process is associated with the reduction of the carbon compound. When hydrogen reacts with triglycerides, different types of reactions can occur, and the different products can be combined (Fig. 20) [127]. The authors also emphasized biomass-to-liquids (BtL), diesel, and bio-synthetic gas (bio-SG) conversion methods for lignocellulosic biomass. Furthermore, algae-based biofuels have gained popularity because of the high lipid content of microalgae, which may lead to biodiesel production. Research is continuing to optimize microalgal culture and harvesting for large-scale biofuel production [83, 136].

### Advanced third-generation biofuels

- Research on microalgae-based biodiesel offers high lipid productivity, environmental versatility, and nonarable land use. However, challenges in terms of cultivation, harvesting, and lipid extraction remain in the laboratory stage [8, 133].

- Sugar conversion to diesel-type biofuels: another area of research involves the conversion of sugars into diesel-type biofuels using biological or chemical catalysts. This approach aims to produce biofuels with properties similar to petroleum-based diesel. Both biological pathways, such as microbial fermentation, and chemical processes, such as catalytic conversion, are being explored to achieve this conversion [106, 134].

### Conversion technologies for biofuel production

#### Advanced biochemical processes

- *Enzymatic hydrolysis.* Progress has been made in the identification and engineering of enzymes that effectively degrade complex carbohydrates in lignocellulosic biomass into fermentable sugars. This involves identifying robust enzymes, optimizing enzyme mixtures, and improving enzyme performance and stability [22].

- *Fermentation.* Research has concentrated on enhancing microbial fermentation methods to produce biofuels. This comprises strain selection and genetic engineering of microbes to improve their capacity to convert carbohydrates into the necessary fuel molecules. Advances in metabolic engineering have permitted the synthesis of biofuels with specific features, such as increased energy density and compatibility with current infrastructure [25, 46].

#### Thermochemical processes

- *Pyrolysis.* Pyrolysis includes heating biomass in the absence of oxygen to produce bio-oil, syngas, and biochar. Advances have concentrated on optimizing pyrolysis variables, such as temperature, residence time, and feedstock properties to maximize the output of desired bio-oil while reducing undesirable byproducts. Catalytic pyrolysis, which uses catalysts to improve the process, has also shown promise for enhancing product selectivity and quality [136].

- *Gasification.* Gasification is a thermochemical process that transforms solid biomass into synthesis gas (syngas), which contains carbon monoxide and hydrogen. Advances in gasification technology have boosted process efficiency and syngas quality, and reduced tar and particulate matter emissions. Syngas may be further processed into different biofuels, including Fischer–Tropsch liquids and hydrogen [161].

- *Hydrothermal liquefaction* (*HTL*). HTL is a process in which biomass is converted into bio-oil at high temperatures and pressures in the presence of water. Recent advances in HTL include the use of catalysts and cosolvents to increase the production and quality of bio-oil, as well as the utilization of diverse feedstocks, such as algae and wet biomass [46].

- *Supercritical fluid conversion.* Supercritical fluid technologies, such as supercritical water or supercritical ethanol, have been studied for the conversion of biomass to biofuels. These reactions work at high temperatures and pressures, modifying the solvent’s characteristics and facilitating the breakdown of biomass components. Advancements in supercritical fluid conversion include process optimization, catalyst development, and integration with other conversion methods [8, 117].

#### Emerging technologies

- *Synthetic biology.* Synthetic biology is the process of creating and engineering biological systems to perform certain purposes, such as biofuel generation, to increase efficiency and yield via pathways, enzyme optimization, and metabolic engineering [136].

- *Microbial electrochemical systems (MES).* MESs use microbial metabolism and electrochemistry to transform organic molecules into biofuels or power. MES research has focused on optimizing electrode materials, microbial consortia, and operating conditions to optimize conversion efficiency and maximize ethanol output [116].

- *Photobiological processes.* Photobiological processes involve the use of photosynthetic microorganisms, such as algae or cyanobacteria, to convert solar energy and CO_2_ into biofuel. Advances in this topic include the genetic engineering of microorganisms to increase their photosynthetic efficiency and lipid synthesis, and the development of photobioreactor technology for large-scale cultivation [9, 145].

Advancements in biofuel conversion technologies aim to improve efficiency, yields, energy efficiency, and sustainability; however, continued research and innovation are needed for widespread biofuel adoption.

## Challenges and opportunities in biofuel production

### Challenges

#### Feedstock availability and sustainability

The availability of sustainable and plentiful feedstocks poses a substantial obstacle to biofuel manufacturing. Competing demands for land, water, and resources from food production, conservation, and biofuel crops can raise concerns about land use change, deforestation, and biodiversity loss. It is critical that biofuel feedstocks are grown in a sustainable and ecologically responsible way [106, 134, 142].

#### Feedstock diversity

The reliance on a restricted variety of feedstocks for biofuel production, such as maize, sugarcane, and soybeans, may result in rivalry with food production and consequent price increases. The development of alternative feedstocks, including as nonfood crops, agricultural leftovers, and waste materials, is critical for minimizing dependency on food-based feedstocks and boosting the sustainability of biofuel production [121, 136].

#### Conversion efficiency

Efficiency issues frequently arise while converting feedstocks into biofuels. Enzymatic hydrolysis, fermentation, and thermochemical conversion are complicated processes that need to be optimized. Increasing conversion efficiency, lowering energy inputs, and limiting byproduct generation are continuing research targets increasing the economic feasibility of biofuel production [71, 92–94].

#### Technological advancements

Continued research and development are required to improve conversion technologies and make them more economically feasible. This involves enhancing enzyme performance and stability, optimizing catalysts, inventing innovative reactor designs, and scaling up processes from laboratory to commercial-scale production [148].

#### Infrastructure and distribution

The current infrastructure and distribution networks were developed largely for fossil fuels and may not be totally compatible with biofuels. Expanding biofuel production involves investments in infrastructure, such as storage, transportation, and fueling stations, to handle biofuel production, distribution, and consumption [108, 157].

### Opportunities

#### Renewable energy transition

Biofuels provide a means to move from fossil fuels to renewable energy sources. Moreover, renewable energy sources can play an important role in lowering greenhouse gas emissions, especially in the transportation sector, when alternative renewable energy sources are limited [92–94, 121].

#### Energy security

Biofuels can help improve energy security by reliance on imported fossil fuels. Domestic production of biofuels from locally accessible feedstocks can help to increase the stability and robustness of energy systems [92–94].

#### Rural development and job creation

Biofuel production may boost rural economies by generating jobs in agriculture, feedstock production, processing, and distribution. It can create new revenue options for farmers and boost rural communities [100, 139].

#### Waste utilization

Biofuel production may use agricultural and forestry wastes, as well as waste materials, diverting them from landfills and mitigating environmental effects. This approach creates an opportunity for waste control and resource optimization [105].

#### Technological innovation

Biofuel production drives technological advancements in feedstock cultivation, conversion technology, and process optimization. Advances in biotechnology, genetic engineering, and synthetic biology can be used to improve biofuel production efficiency and sustainability [92–94, 100].

#### Sustainability criteria and standards

The creation of sustainability certification schemes and standards for biofuels is an opportunity to assure ecologically and socially acceptable production processes [89]. Certifications can assist in the differentiation of biofuels in the market and offer customers confidence in their sustainability [105].

Addressing the obstacles and capitalizing on the potential of biofuel production involves a multidisciplinary strategy that includes scientific research, legislative assistance, corporate sector investment, and public awareness [142]. Collaboration among stakeholders, including researchers, industry, politicians, and environmental organizations, is critical for furthering the sustainable development and implementation of biofuels [148].

## Environmental implications and benefits of biofuels

### Reduction in greenhouse gas emissions

One of the most significant environmental benefits of biofuels is their ability to minimize greenhouse gas (GHG) emissions compared to fossil fuels.

Biofuels are derived from a variety of renewable sources, not limited to biomass alone. While many biofuels are indeed produced from plant-based biomass, which absorbs atmospheric CO_2_ during growth, the category of biofuels encompasses a broader range of feedstocks and production methods.

Biofuels are produced from a diverse array of renewable feedstocks, including but not limited to:

- 1. Plant-based biomass: this includes crops, agricultural residues, and forestry waste, which absorb atmospheric CO_2_ during their growth cycle [117].
- 2. Algae: both microalgae and macroalgae can be used for biofuel production, offering high growth rates and CO_2_ absorption capabilities [107].
- 3. Waste materials: this includes municipal solid waste, food waste, and industrial byproducts, which may not directly absorb CO2 but contribute to sustainability by repurposing waste streams [7].
- 4. Animal fats and used cooking oils: these feedstocks, while not directly absorbing CO_2_, contribute to sustainability by utilizing waste products [48].
- 5. Industrial off-gases: carbon monoxide and hydrogen from industrial processes can be converted to biofuels, indirectly reducing greenhouse gas emissions [37].
- 6. Atmospheric CO_2_: direct air capture (DAC) technology, combined with renewable energy, can produce synthetic biofuels, actively removing CO2 from the atmosphere [82].

The carbon balance and environmental impact of biofuels vary significantly depending on the feedstock and production method. While plant-based and algal biofuels can potentially offer a closed carbon cycle, other feedstocks may provide environmental benefits through waste reduction or by avoiding new greenhouse gas emissions. The sustainability of each biofuel should be assessed on a case-by-case basis, considering factors such as land use change, energy input for production, and overall life cycle emissions [136].

Biofuels are made from renewable biomass, which absorbs atmospheric CO_2_ throughout the growing period. When combusted, biofuels emit CO_2_, but this emission is countered by the carbon absorbed during feedstock cultivation, resulting in net emission reductions [138, 136]. Advanced biofuels, such as cellulosic ethanol and algae-based fuels, have even greater promise for reducing emissions. Cellulosic ethanol has the potential to reduce greenhouse gas emissions by up to 90% [138]). According to the US Department of Energy, biofuels are totally biodegradable, unlike other gasoline additives [142].

### Air quality improvements

Biofuels have the potential to enhance air quality by lowering pollution emissions. Biofuels produce lower amounts of sulfur dioxide, particulate matter, and nitrogen oxides than fossil fuels, all of which have been linked to respiratory and cardiovascular problems. Using biofuels in transportation can help alleviate these health effects and improve local air quality, especially in heavily populated regions [39, 47].

### Land and water resource implications

Biofuel feedstock cultivation has the potential to have a substantial impact on land and water resources. Unsustainable land use practices, such as deforestation or the modification of natural ecosystems, can cause biodiversity loss and carbon emissions [106]. Sustainable biofuel production requires responsible land-use planning, prioritizing degraded land, avoiding ecologically sensitive areas, and implementing water-efficient practices to address water scarcity issues [80]. Biofuel production can have a severe influence on the environment, including deforestation and increased fertilizer and pesticide use. Growing biofuel feedstocks on high-carbon soils, such as peat land, greatly increases GHG emissions. Land use changes can have important environmental impacts, such as soil erosion, nutrient depletion, water consumption, and biodiversity loss, as well as increasing GHG emissions [106].

### Biodiversity considerations

Biofuel feedstock development may have ramifications for biodiversity protection. Clearing land for biofuel crops can result in wildlife habitat loss, ecological disruption, and detrimental implications for biodiversity [137]. Sustainable biofuel production should prioritize the use of nonfood crops, agricultural leftovers, and waste materials to avoid rivalry with food production and put less strain on natural ecosystems. Maintaining biodiversity while boosting biofuel production requires appropriate sourcing procedures, land-use certifications, and the preservation of high-conservation-value places.

## Economic viability and market potential of biofuels

Factors, including production costs, feedstock availability, legislative frameworks, and market dynamics, all have an impact on the economic feasibility and potential of biofuels. There are several important aspects of the economic feasibility and market potential of biofuels.

### Cost competitiveness

The cost of manufacturing biofuels is important for their economic sustainability. It considers feedstock costs, conversion methods, operating expenditures, and distribution infrastructure. Biofuels must be cost competitive with fossil fuels to attract market demand and investment [108, 148]. Advances in technology, scale, and feedstock availability can all assist in reducing production costs and increasing competitiveness [120].

To better understand the cost competitiveness of biofuels compared to conventional fossil fuels, we have compiled recent data from various sources. Table 7 presents a comparison of production costs for different types of biofuels and their fossil fuel counterparts. The cost competitiveness of biofuels remains a dynamic field, with ongoing research and development efforts to reduce production costs and improve efficiency. Future breakthroughs in feedstock development, processing technologies, and scale-up strategies could significantly alter the competitive landscape of biofuels versus fossil fuels.

**Table 7:** Comparison of production costs for biofuels and fossil fuels (International Energy Agency (IEA).2021)

Fuel type	Production cost (USD/liter)	Energy density (MJ/ liter)	Cost per energy unit (USD/GJ)
Conventional gasoline	0.5–0.65	34.2	14.6–19.0
Corn ethanol	0.55–0.75	21.2	25.9–35.4
Sugarcane ethanol	0.45–0.65	21.2	21.2–30.7
Cellulosic ethanol	0.8–1.20	21.2	37.7–56.6
Conventional diesel	0.55–0.70	38.6	14.2–18.1
Biodiesel (soybean)	0.70–0.90	33.3	21.0–27.0
Biodiesel (waste cooking oil)	0.60–0.80	33.3	18.0–24.0
Renewable diesel	0.75–1.00	34.4	21.8–29.1
Advanced biofuels (e.g., algal)	1.2–2.00	35.0	34.3–57.1

To provide a comprehensive overview of the competitiveness of various biofuels, including biojet and bio-oil, we have compiled data from recent studies and industry reports. Table 8 presents a comparison of key parameters for these biofuels and their conventional counterparts.

**Table 8:** Comparative analysis of biofuels and conventional fuels

Fuel type	Production cost (USD/liter)	Energy density (MJ/liter)	GHG emissions (g CO_2_e/MJ)	Compatibility with existing infrastructure
Conventional jet fuel	0.4–0.60	35.3	87.5	High
Biojet (HEFA)	0.80–1.20	34.3	25.0	High
Biojet (FT-SPK)	1.00–1.50	34.3	12.5	High
Conventional Diesel	0.55–0.70	38.6	95.1	High
Biodiesel (FAME)	0.70–0.90	33.3	31.3	Medium
Renewable diesel (HVO)	0.75–1.00	34.4	29.2	High
Conventional gasoline	0.50–0.65	34.2	93.3	High
Bio-oil (fast pyrolysis)	0.60–1.00	16–19	37.2	Low
Ethanol (corn)	0.55–0.75	21.20	57.5	Medium
Ethanol (sugarcane)	0.45–0.65	21.20	24.3	Medium

*HEFA* hydroprocessed esters and fatty acids, *FT-SPK* Fischer–Tropsch synthetic paraffinic kerosene, *FAME* fatty acid methyl esters, *HVO* hydrotreated vegetable oil

It is important to note that the competitiveness of biofuels is not static and can be significantly influenced by factors, such as government policies, technological advancements, and changes in feedstock availability and prices. Ongoing research and development efforts are focused on improving production efficiencies and reducing costs, which could further enhance the competitiveness of biofuels in the future [11].

### Government policies and incentives

Government regulations and incentives are critical in advancing the biofuel business. Mandates, tax credits, subsidies, loan guarantees, and renewable fuel requirements are all possible options. Supportive policies provide a favorable market environment, encourage investment, and provide stability to biofuel producers. The degree and length of governmental assistance can have a substantial influence on the market potential of biofuels [11, 44, 81].

### Fossil fuel prices

Price fluctuations in fossil fuels can have an impact on the market potential of biofuels. When fossil fuel costs rise, biofuels become more competitive and appealing to customers. However, if fossil fuel costs fall dramatically, biofuels may struggle to retain competitiveness [60, 134].

### Market demand and infrastructure

The market potential of biofuels is determined by consumer demand as well as the availability of infrastructure for biofuel production, distribution, and consumption. The transportation industry is an important market for biofuels, and the use of biofuel-compatible cars and fueling infrastructure is critical. A growing public understanding and acceptance of biofuels might also boost market demand [42, 136].

### Feedstock availability and sustainability

The availability of sustainable and plentiful feedstocks at acceptable prices is critical to the economic sustainability of biofuels. Diversifying feedstocks, using waste resources, and improving feedstock yield can all contribute to increasing the market potential of biofuels. Sustainable feedstock production techniques and certification programs can also help with the market adoption of biofuels [106, 142].

### Technological advancements

Advances in conversion technology, such as enhanced enzyme efficiency, process optimization, and production scale, can help biofuels become more economically viable. Technological innovation can assist in lowering production costs, improving output, boosting energy efficiency, and improving the quality of biofuels [43, 134].

### International trade and market dynamics

International trade and market dynamics might influence the market potential of biofuels. Biofuel imports and exports can be affected by different nations' trade policies, taxes, subsidies, and restrictions. Furthermore, market variables such as customer preferences, energy security concerns, and environmental restrictions impact biofuel consumption across locations [20, 103].

It is crucial to remember that the economic feasibility and market potential of biofuels differ by location and are dependent on individual market conditions, legislation, and feedstock availability. As technology advances, costs fall, and regulatory support remains constant, the market for biofuels is projected to expand. However, continued research, innovation, and stakeholder engagement are critical for realizing the full economic potential of biofuels and enabling a long-term transition to renewable energy sources [60].

## Policy and regulatory frameworks for biofuel development

Policy and regulatory frameworks are critical for encouraging the development and implementation of biofuels. They establish rules, incentives, and standards to help the sector and guarantee that biofuels are produced and used sustainably. The following are some important components of the policy and regulatory frameworks for biofuel development.

### Renewable fuel standards (RFS) and blending targets

Many nations have enacted renewable fuel standards or blending goals, which require a particular number of biofuels as transportation fuels. These measures increase the market demand for biofuels while also providing long-term stability for producers. Blending objectives can be based on volume (e.g., a certain amount of biofuel to be blended) or percentage (e.g., a specified proportion of biofuels in the total fuel mix) [53, 60].

### Feedstock regulations

Policies and regulations handle feedstock-related concerns such as land usage, sustainability standards, and certification. These systems guarantee that biofuel feedstocks are supplied sustainably, avoiding deforestation, biodiversity loss, and negative social consequences. Certification programs, such as the Roundtable on Sustainable Biomaterials (RSB) and the International Sustainability and Carbon Certification (ISCC), provide requirements for sustainable feedstock production [101, 172].

### Financial incentives

Governments frequently offer financial incentives to encourage biofuel production and usage. Tax credits, grants, loan guarantees, and subsidies may be used to offset the greater costs of producing biofuels in comparison to fossil fuels. Financial incentives serve to attract investment, decrease market obstacles, and boost the use of biofuels [122, 172].

### Research and development support

Governments may sponsor biofuel-related research and development efforts. This assistance encourages technical innovation, efficiency gains, and the development of new feedstocks. Research funding, cooperation with academic institutions, and public‒private partnerships are prominent means of encouraging innovation in biofuel technology [15].

### Import and export policies

International trade rules and regulations can influence the biofuel industry. Tariffs, quotas, and trade agreements influence the import and export of biofuels and feedstocks. Governments may set trade restrictions to safeguard domestic businesses or develop preferential trade agreements to encourage biofuel exports [66, 131].

### Environmental regulations

Environmental laws, such as emissions and air quality requirements, have the potential to affect biofuel consumption. Biofuels are generally preferred over fossil fuels because they emit fewer greenhouse gases. Governments may set emission-reduction objectives or carbon pricing schemes to encourage the use of biofuels [59, 113].

### Infrastructure support

Governments may help fund the construction of biofuel infrastructure, including filling stations, storage facilities, and distribution networks. Funding for infrastructure expansion and upgrading existing infrastructure to handle biofuels helps overcome market obstacles and increase biofuel use [53, 130, 150, 163].

### Public awareness and education

Specific policy and regulatory frameworks for biofuel development differ by country and area, reflecting local goals, resources, and market conditions. Governments frequently work with industry players, research institutes, and environmental groups to develop successful policies that balance economic development, energy security, and sustainability objectives. Policy frameworks must be continuously evaluated and updated to respond to changing technology, market dynamics, and environmental concerns [60, 63].

## Future prospects and research directions

### Technological advancements and innovation

The development of new technologies and innovative solutions can assist in increasing the efficiency and sustainability of biofuel production. For example, researchers are investigating the use of nanotechnology to increase biofuel production [158], improve biofuel production efficiencies through the use of new catalysts, and biorefinery designs, utilize biotechnology such as genetic engineering to increase energy crop yields, and continue to reduce the costs of cellulosic ethanol and other advanced biofuel technologies [49, 130].

### Integration of biofuels with other renewable energy sources

Combining biofuels with other renewable energy sources (hybrid systems), such as wind and solar power and hydropower, can contribute to a more sustainable and dependable energy system [78, 150]. Using renewable electricity in industry processes. Potential synergies with integrated biorefineries that produce renewable chemicals and minerals [130, 158].

### Exploration of new feedstock options

Researchers are investigating novel biofuel feedstock options, such as algae, camelina, municipal solid waste, and other specialized feedstocks, to help reduce the environmental impact of biofuel production (Mat et al., 2020; [71]. Marginal or degraded areas should be utilized to minimize competition with food production. Bioengineering crops such as sorghum, switchgrass, and oilseed crops are used for biofuel production [136, 150, 158].

### Addressing sustainability challenges and optimizing production processes

It is critical to address the sustainability issues connected with biofuel production, such as land use changes and water consumption, as well as optimize production techniques to reduce environmental effects [71, 106], implementing environmentally friendly agricultural methods for energy crop cultivation, and concentrating on waste/residue supply networks. A continuing lifetime evaluation study is needed to compare technologies/feedstocks and priority improvements, regional optimization of biofuel crop choices and biorefinery sites [106, 148].

## Conclusions

Biofuels have the potential to significantly reduce dependency on fossil fuels and their associated greenhouse gas emissions, playing an important role in the global energy transition. First-generation biofuels made from food crops have been criticized for their sustainability, while advanced biofuels promise to harness the advantages of bioenergy while minimizing unforeseen effects. Cellulosic ethanol from waste biomass and promising technologies such as algae biofuels are becoming increasingly commercially viable and might be generated on a large scale in a sustainable manner. Additional research and development are still necessary to boost production and efficiency while lowering expenses. However, with supporting policies, advanced biofuels have the potential to spread as an emission-reduction option that is compatible with existing cars and infrastructure.

The adaptability of biofuels enables them to blend with emerging energy systems that rely more on solar, wind, and other renewables. Waste carbon emissions can even be repurposed using tailored biofuel processes. Furthermore, integrated biorefinery systems have the potential for synergistic effects on the production of biofuels as well as bio-based chemicals and materials. With further innovation and appropriate deployment, biofuels can play an important role in establishing long-term low-carbon economies while avoiding the difficulties previously encountered. The distinct features of liquid biofuels position them as pioneering agents for greener transportation systems in the future. While decreasing fossil fuel consumption is still critical in the battle against climate change, biofuels provide one way to fuel the Earth's constant energy flows in a brighter future.

While progress has been made in terms of biofuels in recent years, realizing their full potential will require continued efforts across industries. More research is needed to increase production, improve sustainability, and enable large-scale commercialization. Public and commercial players should continue to develop biofuel technologies such as cellulosic ethanol production and algal biofuel systems. It is equally crucial to continue developing sustainable strategies for feedstock cultivation and supply networks. Land use concerns must remain a top emphasis. Policymakers must encourage best practices while fostering greater innovation. International collaboration can help to reconcile global competitiveness with responsible manufacturing.

## Data Availability

No datasets were generated or analyzed during the current study.
